# A molecular signature of lung cancer: potential biomarkers for adenocarcinoma and squamous cell carcinoma

**DOI:** 10.18632/oncotarget.22298

**Published:** 2017-11-06

**Authors:** Varda Shoshan-Barmatz, Yael Bishitz, Avijit Paul, Yakov Krelin, Itay Nakdimon, Nir Peled, Avia Lavon, Elina Rudoy-Zilberman, Yael Refaely

**Affiliations:** ^1^ Department of Life Sciences and The National Institute for Biotechnology in the Negev, Ben-Gurion University of the Negev, Beer-Sheva, Israel; ^2^ Thoracic Cancer Unit and The Center for Precision Cancer Care, Davidoff Cancer Center, Petach Tiqwa, Israel; ^3^ Department of Cardio-Thoracic Surgery, Soroka University Medical Center and The Faculty of Health Sciences, Ben-Gurion University of the Negev, Beer-Sheva, Israel

**Keywords:** biomarkers, lung cancer, adenocarcinoma and squamous cell carcinoma

## Abstract

Adenocarcinoma (AC) and squamous cell carcinoma (SCC), sub-types of non-small cell lung cancer (NSCLC), both present unique features at the genome, epigenome, transcriptome and proteome levels, as well as shared clinical and histopathological characteristics, but differ in terms of treatment. To ensure proper treatment, one must be able to distinguish between these sub-types. Here, we identify novel biomarker proteins in NSCLC, allowing for distinguishing between the AC and SCC sub-types. Proteomics analysis distinguished between healthy and tumor tissues, with the expression level of 1,494 proteins being altered, 378 of which showed a ≥|100|-fold change. Enrichment of proteins related to protein synthesis and degradation, and of proteins associated with mitochondria, metabolism, and apoptosis, was found. Network analysis defined groups of proteins, such as those associated with cell metabolic processes or with fatty acid/lipid metabolism and transport. Several biomarkers that enable for distinguishing between AC and SCC were identified here for the first time, and together with previous reports confirmed here, led us to propose a list of proteins differentially expressed in SCC and AC. Some of these biomarkers are clear signatures for AC or SCC and four of them are secreted proteins. The presence of the mitochondrial protein SMAC/Diablo in the nucleus was found to be a signature for SCC. Precise diagnosis of AC and SCC is essential for selecting appropriate treatment and thus, increasing patient life expectancy. Finally, the search for drugs that target some of these biomarkers may lead to new treatments for lung cancer.

## INTRODUCTION

Non-small cell lung cancer (NSCLC) is the most prevalent form of lung cancer and represents the leading cause of cancer deaths worldwide in both men and women.[1]. Because the majority of diagnosed NSCLC patients are in advanced stages of the disease, overall survival after standard treatment with platinum-based chemotherapy, radiation, and/or surgery remains less than 12 months [2]. Median overall survival can, however, be increased by novel strategies implementing immunotherapies in different combinations [2] or if a driver mutation exists, then survival can be increased to four years by targeted tyrosine kinase inhibitory therapy [3]. NSCLC can divided into a number of sub-types, with the two main sub-types being adenocarcinoma (AC) and squamous cell carcinoma (SCC), together accounting for the vast majority of NSCLS cases (representing almost 80% of primary lung cancer cases [4]) and being responsible for 30% of all cancer deaths. Specifically, AC is the most prevalent sub-type of lung cancer in non-smokers [5], and constitutes approximately 50% of all cases of lung cancer [6]. In AC, the tumor develops from glandular cells of the lungs that are responsible for producing mucin and surfactants. SCC, which constitutes approximately 30% of NSCLC cases, usually develops in central areas of the bronchi of the lung and is closely connected with smoking [7]. Although these two NSCLC sub-types have both unique and shared clinical presentations and histopathological characteristics, the treatment strategy may differ significantly. To insure proper treatment, the ability to distinguish the two NSCLC sub-types during diagnosis is crucial [1, 8]. Current histological discrimination is based on tissue availability, where in ∼ 15-20% of cases, the tissue is exhausted without having being useful for defining final histology result, while as many as 7.2% of cases are poorly differentiated and present not otherwise specified NSCLC.

Lung cancer, as many other cancers, develops via a multistep process of tumor biogenesis involving accumulation of inherited or acquired genetic abnormalities [9]. These can be detected by deep sequencing methods [10], although this is complicated by the heterogeneity and complexity of malignant tumors [11]. Other cancer-associated changes are not mutation–related but rather appear as increases or decreases in protein expression or as differential post-translational modification of marker proteins [12]. Thus, biomarkers other than mutations should be identified and explored as early markers of the disease, as indicators of the disease state, and as predictive and prognostic gauges of treatment effectiveness [12].

Recent efforts have focused on changes that occur within the genome, epigenome, transcriptome, and proteome in lung AC and SCC that could serve to distinguish between these two NSCLC sub-types [7]. Currently, about 17 biomarkers were reported to be differentially expressed in AC and SCC (Supplementary Table 1). Among these are microRNAs, with miR21 being detected in AC, and miR205 being seen in SCC [7]. Similarly, TTF1 (thyroid transcription factor 1), NAPSA (napsin A) and CD141 (thrombomodulin) were found to be highly expressed in AC, as compared to SCC, while high expression levels of TP63 (tumor protein 63) and its isoform p40 (ΔNp63) were reported for SCC [13]. Thus, there are presently 12 biomarkers for AC and 5 biomarkers for SCC, 4 markers of which are used in the clinic to distinguish between these two sub-types and 6 of which are used to direct targeted therapy (Supplementary Table 1).

In this study, using cancer tissue arrays of lung tumors and samples from the lungs of cancer patients, together with mass spectroscopy, immunohistochemistry, immunoblotting, quantitative PCR (qPCR) and bioinformatics tools, we explored proteins that are differentially expressed in lung cancer tumors, relative to healthy tissue. We identified several metabolism- and apoptosis-related proteins, as well as other proteins, that could serve as potential lung cancer biomarkers for AC and SCC, of which AKR1B10, NPC2, GGH and AZGP1 are secreted proteins. Thus, we propose a biomarker signature that could potentially enable early diagnosis of NSCLC, and distinguish between AC and SCC. These biomarkers can also serve as a predictor of treatment effectiveness, and offer potential new targets for therapy development.

## RESULTS

Tumor and healthy samples from the same lung cancer patients were analyzed using LC-HR MS/MS, immunohistochemistry and immunoblotting to explore potential novel disease markers and to distinguish between AC and SCC. Healthy and tumor samples were taken from the same lung, allowing for internal controls, resulting in the identification of 1,494 differentially expressed proteins. In this manner, many potential biomarkers for AC and some for SCC were identified. The results thus offer novel biomarkers for AC and, particularly, for SCC diagnosis.

### Mass spectrometry analysis of the protein profiles of healthy and tumor tissues from NSCLC patients

**Figure 1:**
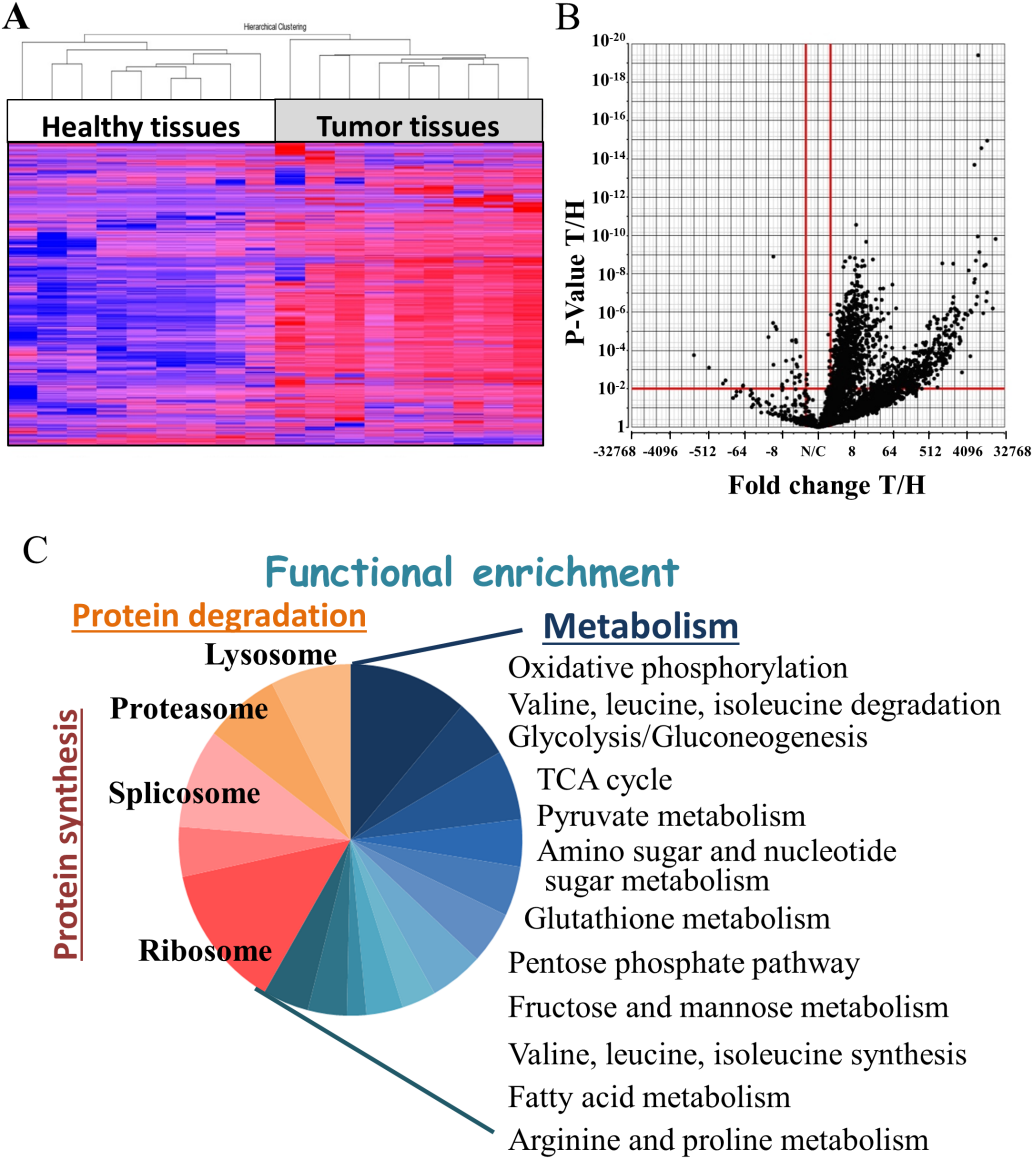
Statistical and functional analysis of protein expression in samples obtained from healthy and tumor lung samples of lung cancer patients Protein expression profiles obtained from healthy and tumor lung samples of lung adenocarcinoma (AC) patients were analyzed by LC-HR MS/MS, as described in Materials and Methods. **(A)** Hierarchical clustering based on the expression pattern of all 2,959 detected proteins with at least 2 unique peptide are presented. Healthy (white) and tumor (grey) samples are indicated. The color scale of the standardized expression values is shown on the right. **(B)** Volcano plot representing the fold change (X axis) and FDR (Y axis) values for each identified protein. Vertical red lines indicate a fold change > 2 or < -2 and the horizontal red line indicates a p-value < 0.05. 1,494 proteins passed these thresholds. **(C)** Significantly enriched functional groups in the proteins showing changed expression, based on the David Gene Ontology system.

To identify proteins showing modified expression levels in NSCLC tumor tissues, relative to healthy tissues, nine samples of cancer and healthy tissues were collected from the same lung of NSCLC patients and subjected to LC-HR MS/MS analysis. Hierarchical clustering based on the expression pattern of all detected proteins clearly allowed for distinguishing between healthy and tumor tissues (Figure 1A), with the expression level of 1,494 proteins being changed (fold change (FC) ≥|2|, with a false discovery rate (FDR) < 0.05, of which 378 proteins showed a FC ≥|100|) (Figure 1B). The up- and down-regulated proteins were further divided into two clusters, based on the combination of FC and p-value, due to some of the proteins being “absent” from some of the samples.

Next, functional analysis of the proteins differentially expressed between cancerous and healthy lung tissues was performed using the DAVID Gene ontology databases [14, 15]. Such analysis revealed enrichment of proteins related to protein synthesis and degradation, and in particular, of proteins assigned roles in metabolism and to the mitochondria (Figure 1C) (Supplementary Table 5).

### Modified expression of metabolism- and apoptosis-related proteins

**Figure 2:**
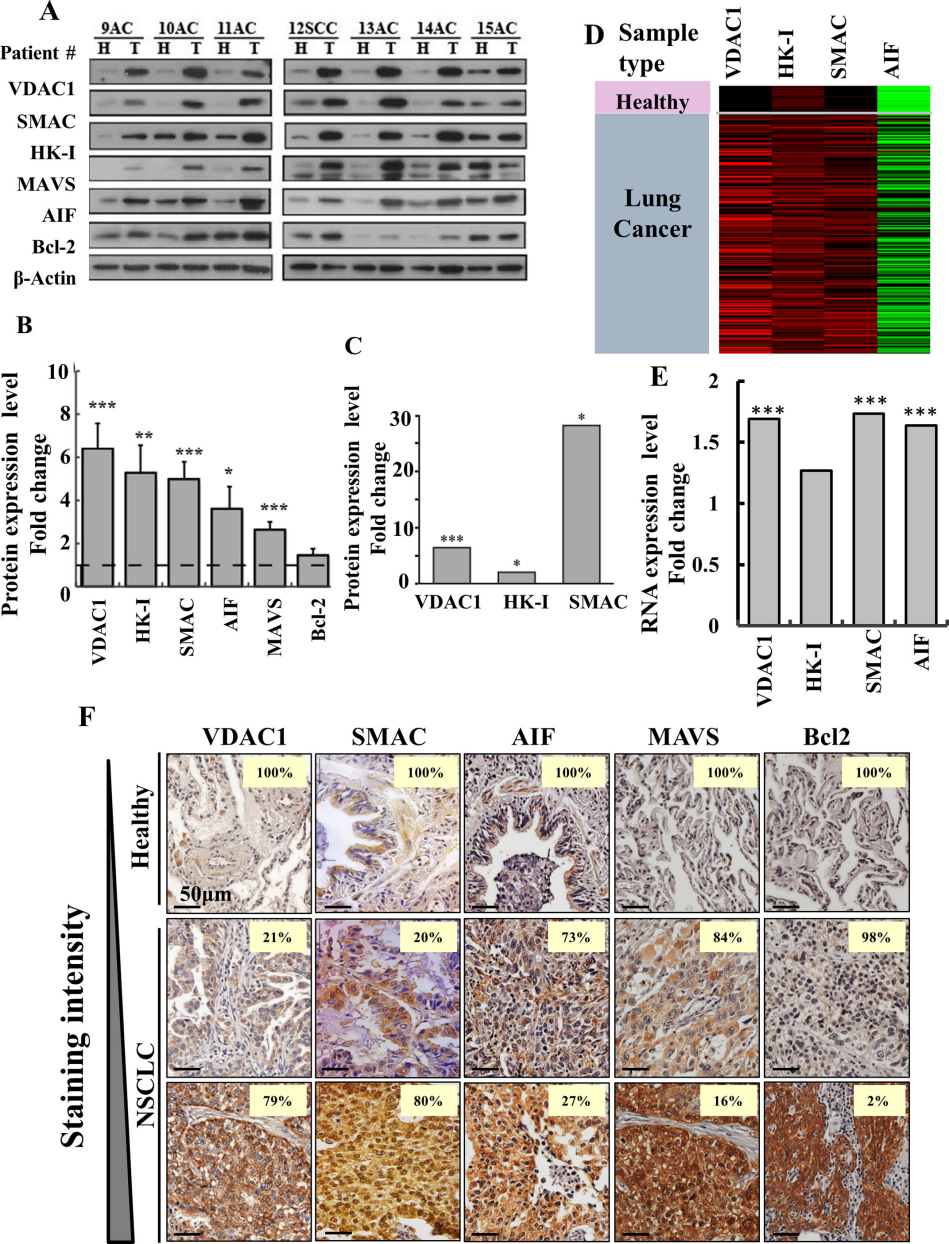
Over-expression of VDAC1 and other apoptosis- and energy-related proteins in lung cancer patients **(A)** Representative immunoblots of tissue lysates of tumor (T) and healthy (H) lung tissues derived from lung cancer patients probed with antibodies directed against VDAC1, SMAC, HK-I, MAVS, AIF and Bcl-2. **(B)** Quantitative analysis of VDAC1 (37 patients, FC=6.2, p-value=5×10^−5^); SMAC (37 patients, FC=5, p-value=3.4×10^−5^); HK-I, (33 patients, FC=5.3, p-value=5.3×10^−3^); MAVS (22 patients, FC=2.6, p-value=1.5×10^−4^); AIF (35 patients, FC=3.5, p-value=1.7×10^−2^), and Bcl-2 (22 patients, FC=1.5, p-value=1.4×10^−1^) are presented as the mean ± SD. **(C)** LC-HR MS/MS data for VDAC1, HK1 and SMAC. A difference between healthy and tumor tissues was considered statistically significant when P < 0.001 (^***^), P < 0.01 (^**^), P< 0.05 (^*^), as determined by the Mann-Whitney test for the immunoblots and a two-way t-test for the LC-HR MS/MS data. **(D)** Heat map showing gene expression based on RNAseq UCSC XENA data of VDAC1, HK-I, SMAC and AIF. The gene expression profiles obtained from healthy (n=110) and tumor lung samples (n=1,017) of lung cancer patients are publicly available (TCGA lung cancer dataset, detailed in Supplementary Data). **(E)** Quantitative analysis of the RNAseq data. **(F)** Over-expression of VDAC1, SMAC, AIF, MAVS and Bcl-2 in lung cancer patients. Representative IHC staining for VDAC1, SMAC AIF, MAVS and Bcl-2 of healthy (n=10) and lung cancer (n=70) tissue samples from tissue microarray slides (US Biomax). The number on each image represents the percentage of patient samples that stained at the relative intensity presented by a gradient line on the left.

As modified metabolism and the development of anti-apoptotic mechanisms are hallmarks of cancer, we decided to focus on several proteins associated with these hallmarks (Figure 2). Samples of cancer and healthy tissues from the same lung of NSCLC patients were analyzed by immunoblotting using specific antibodies to assess levels of the voltage-dependent anion channel 1 (VDAC1), hexokinase I (HK-I), SMAC/Diablo (SMAC), apoptosis inducing factor (AIF), mitochondrial anti-viral signaling (MAVS) and Bcl2. All of these proteins, with the exception of Bcl2, were significantly over-expressed (3- to 6-fold) in cancerous tissues, as compared to healthy tissues obtained from the same NSCLC patient (Figure 2A, 2B). LC-HR-MS/MS further confirmed that expression levels of VDAC1, HK-I and SMAC were highly increased in the cancer tissues (Figure 2C). The RNA expression levels of VDAC1, HK-I, SMAC and AIF showed a similar trend, although expression at the RNA level was lower, as revealed by the RNAseq UCSC XENA data (Figure 2D, 2E).

The expression levels of VDAC1, SMAC, AIF, HK-I, MAVS and Bcl2 was also analyzed by IHC in tissue microarrays comprising healthy and NSCLC-derived samples. The numbers of patient samples showing staining at the indicated intensity, represented as a percentage of the total number of sections analyzed, are shown (Figure 2F). All proteins were highly expressed in the tumor tissue. Thus, although SMAC and AIF are pro-apoptotic proteins, they are over-expressed in tumor tissue, as will be discussed.

**Figure 3:**
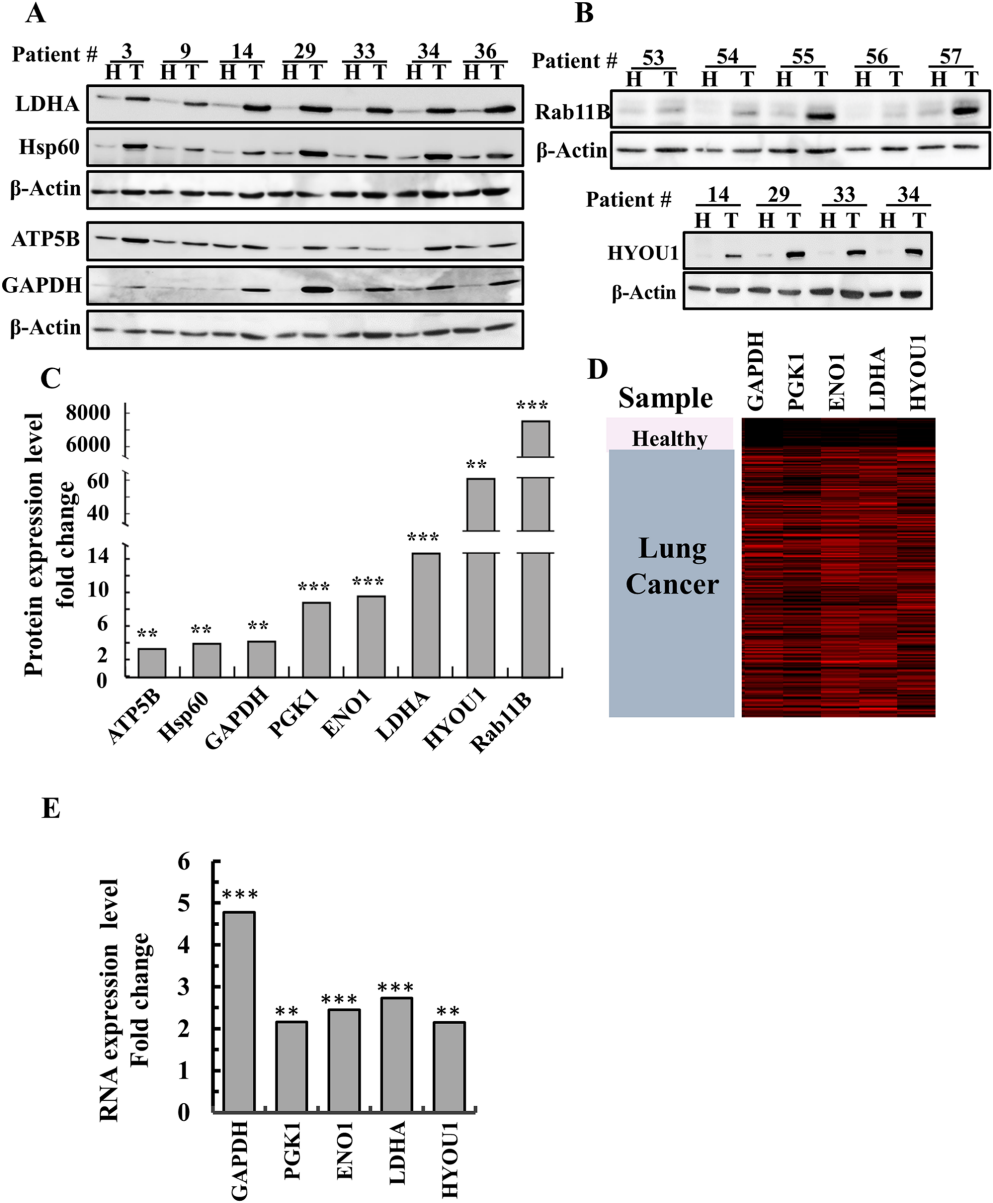
Over-expression of newly identified proteins in lung cancer patients **(A, B)** Representative immunoblots of tissue lysates of tumor (T) and healthy (H) lung tissues derived from lung cancer patients probed with antibodies directed against HYOU1 (ORP150), LDHA, HSPD1 (Hsp60), ATP5B, GAPDH and Rab11b. **(C)** Quantitative analysis of LC-HR MS/MS data. A difference between healthy and tumor tissues was considered statistically significant when P < 0.001 (^***^), P< 0.01 (^**^), as determined by a two-way t-test for the LC-HR MS/MS data. **(D)** Heat map showing gene expression based on RNAseq UCSC XENA data of GAPDH, PGK1, ENO1, LDHA and HYOU1. The gene expression profiles obtained from healthy (n=110) and tumor lung samples (n=1,017) of lung cancer patients (detailed in Materials and Methods). **(E)** Quantitative analysis of the RNAseq data.

Other metabolism-related proteins, such as lactate dehydrogenase (LDHA), the ATP synthase subunit 5B (ATP5B), the glycolysis enzyme glyceraldehyde 3 phosphate dehydrogenase (GAPDH), phosphoglycerate kinase 1, (PGK1) and enolase-1 (ENO1), were also highly expressed (up to 14-fold higher) in the tumor tissues, as determined by LC-HR-MS/MS analysis (Figure 3A, 3C, 3D, 3E).

These results point to the significance of reprogrammed metabolism and apoptosis avoidance in lung cancer, and may point to potential novel treatment targets.

### Identification of novel bio-markers of lung cancer

LC-HR-MS/MS analysis data revealed many other proteins that were differentially expressed in the NSCLC tumors (Supplementary Table 5). The proteins with the most significant changes in expression in the tumors are listed in Supplementary Table 5, along with their proposed function and relation to cancer. These include the neuropilin 2 isoform (NRF2), Ras-related protein Rab11B (Rab11B), a member of the Ras superfamily of small GTP-binding proteins, HYOU1 (ORP150), which plays a pivotal role in cytoprotective cellular mechanisms triggered by oxygen deprivation, and the heat-shock protein HSPD1 (HSP60). These findings were confirmed by immunoblot analysis, RNAseq UCSC XENA data and q-PCR (Figure 3).

**Figure 4:**
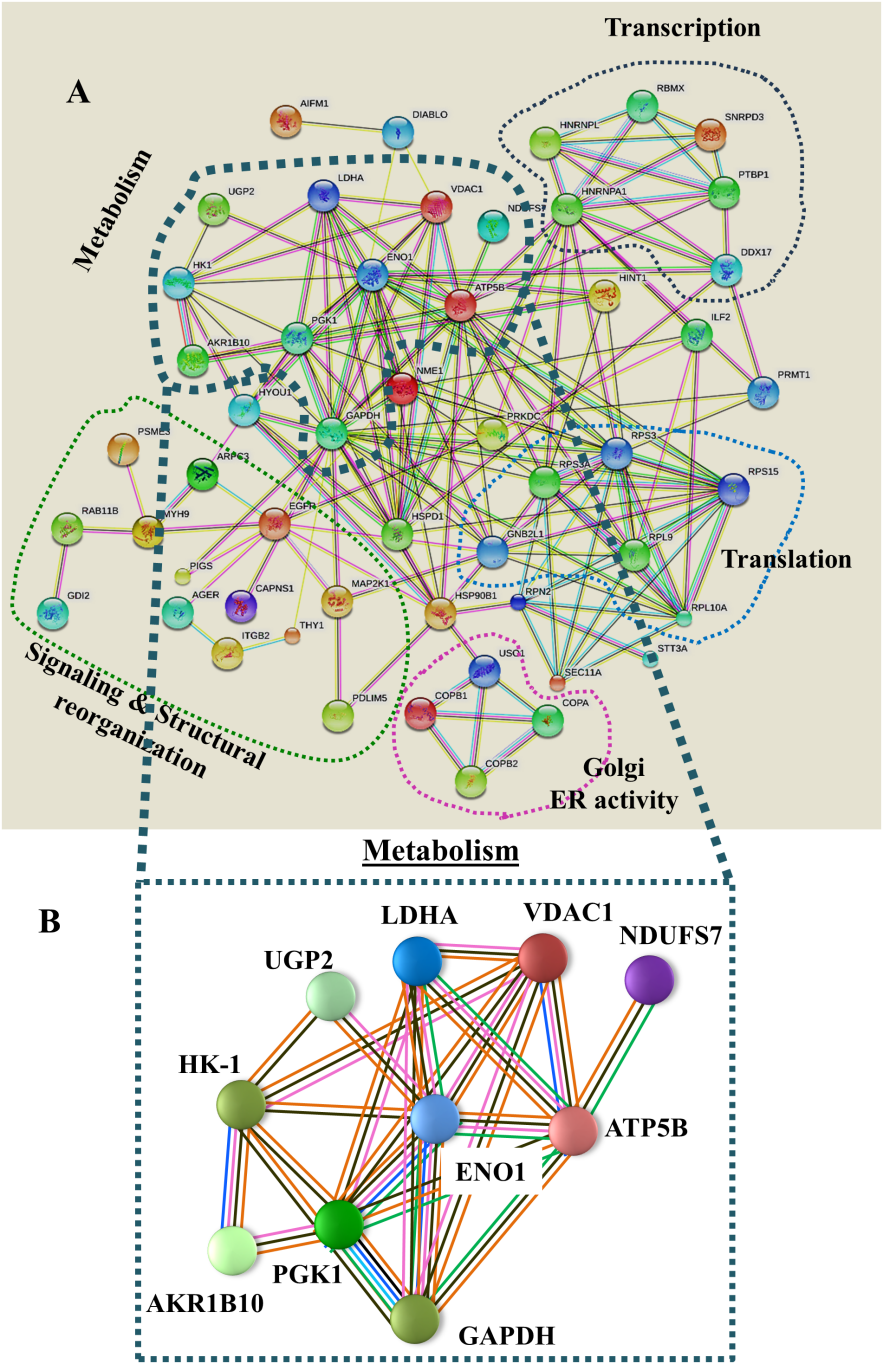
Network analysis of proteins that were differentially expressed in lung cancer patients **(A)** Networks of proteins that were differentially expressed in lung cancer patients (Supplementary Table 5) were produced using https://string-db.org, Experimentally determined protein-protein interactions are presented by lines. These interactions include protein interactions predicted by curated databases, protein co-expression, gene proximity, and as defined by genetic clustering (e.g. sharing expression regulation, such as by epigenetic modifications, physical interaction with the nuclear lamina, co-expression, etc.). Groups of proteins sharing similar or related functions are circled by colored lines with their interactions are presented. **(B)** A metabolism-related group is presented. Pink lines represent experimentally determined interactions, blue lines show protein interactions predicted by curated databases, black lines represent protein co-expression, solid green lines represent neighboring genes, as defined above, and orange lines represent text mining results.

Network analysis (Figure 4) of the proteins identified here by proteomics demonstrated that most of these proteins interact at several levels, with metabolic process-related proteins being central. These interactions include common functionalities associated with cell metabolism, and involve direct physical interaction with each other. Indeed, many are co-expressed and can be defined as being encoded by a cluster of genes that is regulated by epigenetic modification.

### Proteins differentially expressed in AC and SCC

**Figure 5:**
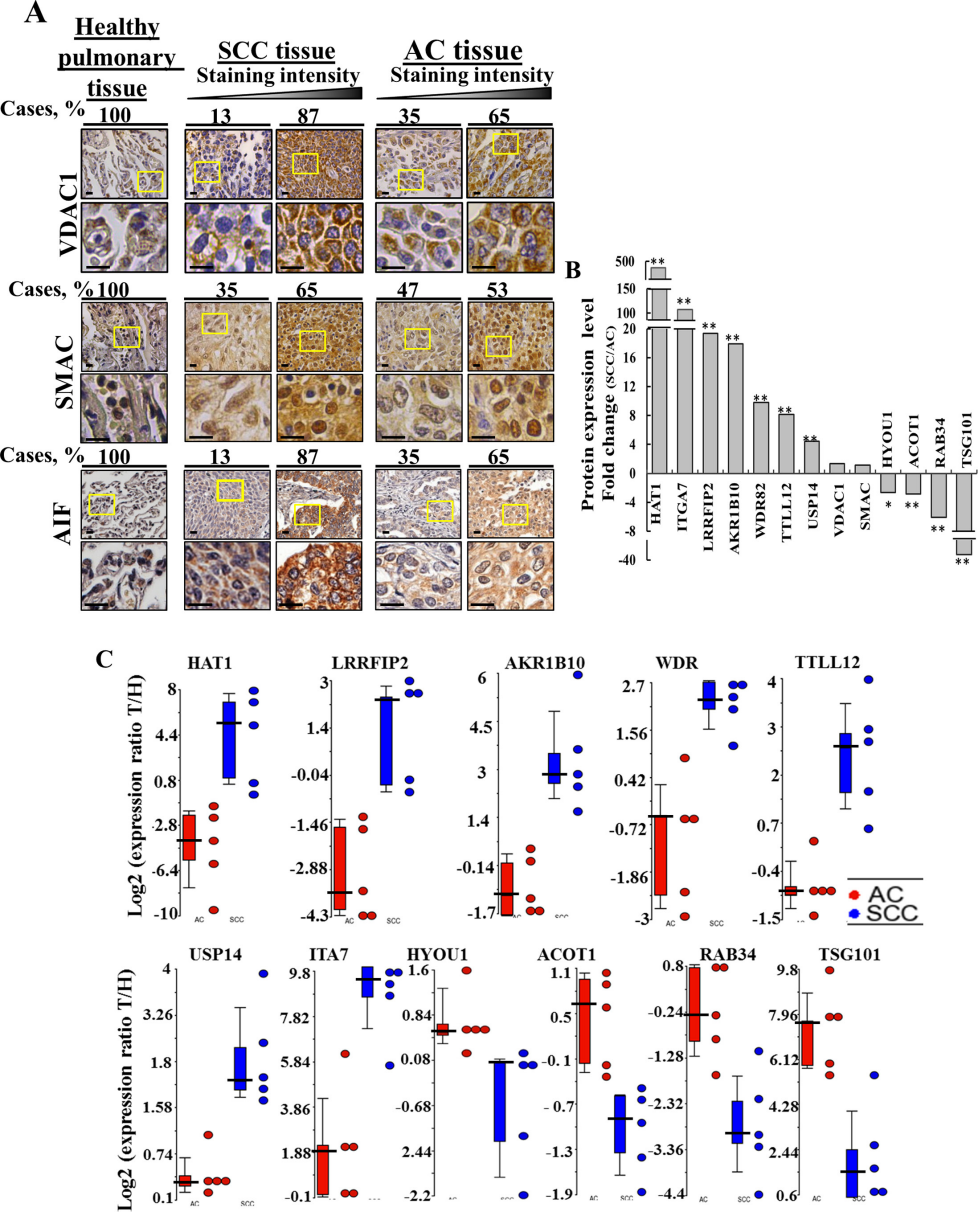
Proteins differentially expressed in AC and SCC **(A)** Tissue array slides (US Biomax) containing human Healthy lung tissue (n=10), lung SCC tissue (n=31) and lung AC tissue (n=21) were stained for VDAC1 and AIF while healthy lung tissue (n=20), lung SCC tissue (n=72) or lung AC tissue (n=72) were stained for SMAC, as described in Supplementary Data. The percentages of patient sample-derived sections stained at the intensity indicated are shown (Cases, %). **(B)** LC-HR MS/MS data were used to identify proteins that can serve to distinguish between AC and SCC. A difference between AC and SCC groups was considered statistically significant when P< 0.05 (^*^), P< 0.01 (^**^) or P < 0.001 (^***^) as determined by the Mann-Whitney test. **(C)** Scatter plots display the expression levels (from LC-HR MS/MS data) of TTLL12, AKR1B10, USP14, LRRF2, HAT1, TSG101, WDR, ACOT1 as the log2 ratio of healthy to tumor for AC and SCC after zero intensities were replaced with 1. To eliminate inflated ratios caused by division by 1, log2 ratios larger than 10 (∼1000 fold in linear scale) were replaced by 10. For each protein, a t-test was carried out between the log2 ratios of the SCC patients and the log2 ratios of the AC patients. A total of 10 proteins had nominal p-value < 0.01. No protein had FDR adjusted p-value < 0.1. Statistics were calculated with GraphPad Prism software. Horizontal lines represent mean values for each group.

Analysis of lung tissue microarrays for VDAC1 and AIF (10 healthy, 31 SCC and 21 AC) and for SMAC (20 healthy, 72 SCC and 72 AC) expression levels by IHC staining using specific antibodies revealed high expression of these proteins in lung cancer, as compared to healthy tissue (Figure 5A). Quantitation of the IHC results, presented as the number of patient samples showing staining at the indicated intensity and represented as a percentage of the total number of sections analyzed, showed that VDAC1, SMAC and AIF expression levels were higher in SCC than in AC (Figure 5A).

Next, cancer and healthy tissues samples from the lung of five of each AC and SCC patients were subjected to LC-HR-MS/MS analysis. The expression levels of 2,959 proteins were up- or down-regulated in the cancer tissues, relative to their expression in the corresponding healthy tissue, with the changes in expression of 1,513 proteins being significant. As expected, many of these proteins overlapped with those proteins found to be differentially expressed in the study presented in Figure 1 and Supplementary Table 5.

Next, those proteins differentially expressed between AC and SCC and which showed the highest change in expression levels (p-value < 0.01) between the two NSCLC sub-types were selected and the fold change of expression in the tumor, relative to the healthy tissue, was calculated and presented as the SCC/AC ratio for each protein (Figure 5B). Assessing the SCC/AC ratios revealed that HAT1, ITGA7, LRRFIP2, AKR1B10 (secreted), WDR82, TTLL12, USP14 were highly over-expressed (up to 500-fold) in SCC, as were VDAC1 and SMAC, albeit to a lower extent, while HYOU1, ACOT1, RAB34, TSG101 showed higher expression in AC. These and other proteins showing significant changes in expression levels between AC and SCC are presented in Supplementary Table 6, along with proposed function and relation to cancer, including the lung cancer sub-type. The expression of these proteins in the different patient samples is presented as scatter plots of the log2 ratio of healthy to tumor for AC and SCC (Figure 5C).

**Figure 6:**
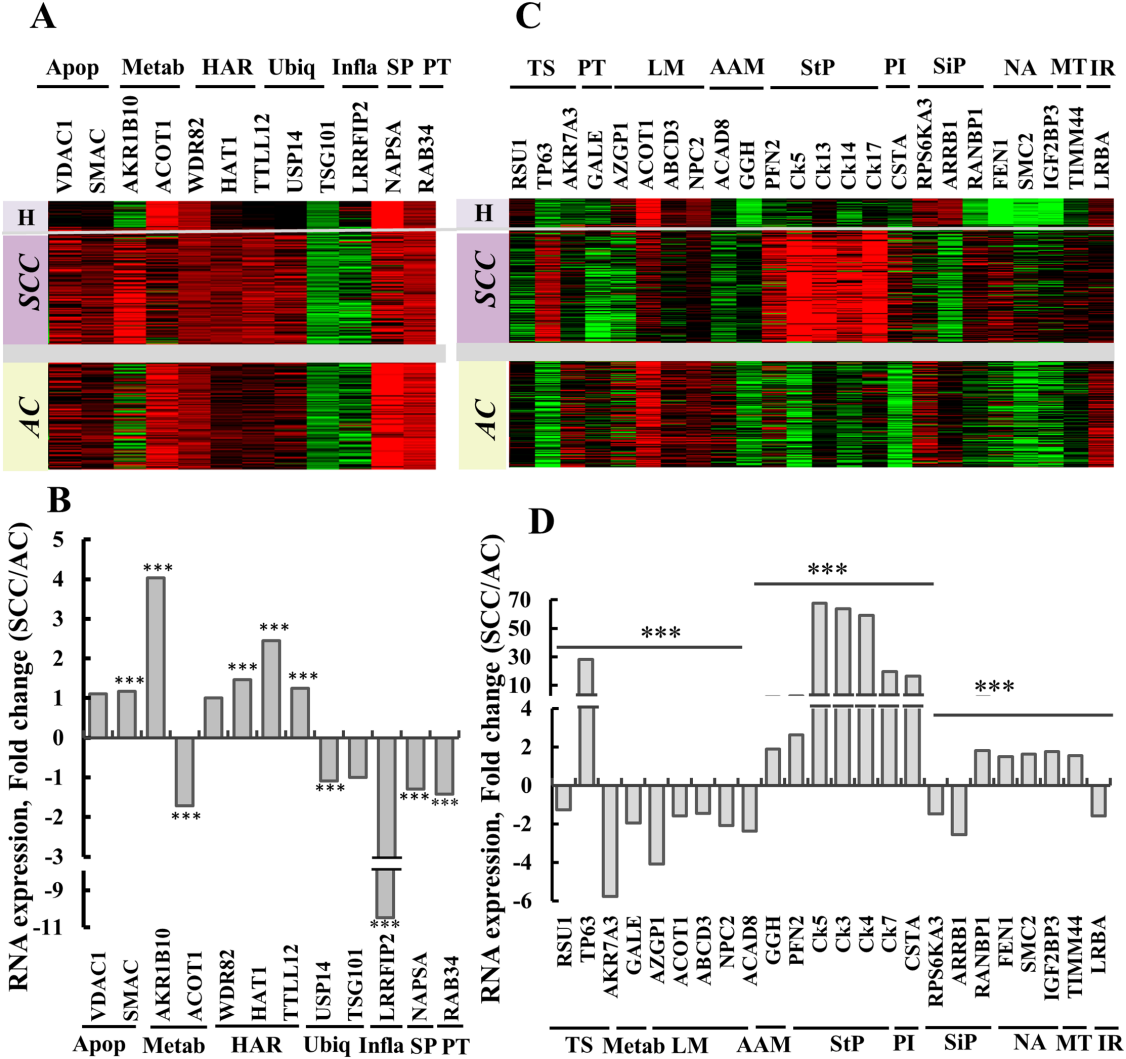
Gene expression as determined by RNAseq of potential protein markers in lung cancer patients Expression patterns of twelve selected genes (Supplementary Table 5), previously proposed [13] or identified in the current study, proposed to distinguish between AC and SCC. **(A)** Gene expression, heat map. Sample type (lung cancer, n=1017, or healthy tissue, n=110) and histological type (SCC, n=527, or AC, n=364), with relative gene expression levels, are presented, with red indicating high, black indicating medium and green indicating low expression levels. **(B)** RNAseq data imported from TCGA was subjected to quantitative analysis using a t-test. The ratio of the expression of the proteins in (A) SCC/AC is presented, and is considered statistically significant when P < 0.001 (^***^). The proteins were grouped according to function as: Apop, apoptosis; Metab, metabolism; HAR, histone activity regulation; Ubiq, ubiquitination; Inflam, Inflammatory response; SP, Surfactant production; PT, protein transport. **(C)** Expression pattern of 24 selected genes (Supplementary Table 6), showing differential expression between AC and SCC based on proteomics data, were analyzed in the RNASeq dataset as presented in (A) and are presented as a gene expression map. Functional groups are indicated: TS, tumor suppressor; Metab, galactose metabolism; LM, lipid metabolism; AAM, amino acid metabolism; StP, structural proteins; PI, proteinase inhibitor; SiP, signaling pathway; NA, nuclear activity; MT, mitochondrial translocase and IR, immune response. **(D)** Quantitative analysis of RNAseq data from (C) carried out as in (B).

Next, we analyzed the expression of the thirteen proteins showing significantly differential expression (MS/MS data, Figure 5B), and of NAPSA (previously proposed for distinguishing between AC and SCC) using RNAseq (UCSC XENA, n=1,129) on tissues obtained from healthy and lung cancer patients (Figure 6A, 6B). Such analysis revealed that ACOT1, RAB34, TSG101, NAPSA expression levels were lower in SCC than in AC. In contrast, the proteins AKR1B10 (secreted), HAT1 and TTLL12 showed higher expression in SCC than in AC. Thus, these results are in agreement with the proteomics data (Figure 5B, 5C), and allow us to propose the use of these proteins as markers to distinguish between AC and SCC.

In an attempt to identify other proteins from the proteomics data, showing large differences in expression levels between AC and SCC were addressed using RNAseq UCSC XENA data (Figure 6C, 6D). Levels of RNA for TP63, GGH (secreted), Ck5, Ck13, Ck14, Ck17, CSTA, RANBP1 and FEN1 were increased in SCC, relative to AC, while RSU1, AKR7A3, GALE, AZGP1 (secreted), NPC2 (secreted), ACAD8, RPS6KA3, ARRB1 and LRBA RNA levels showed the opposite trend, namely higher expression in AC, relative to SCC. The functions of the products of these genes and their relation to AC or SCC are listed in Supplementary Table 7.

### Expression of proteins associated with survival rates in AC and SCC

**Figure 7:**
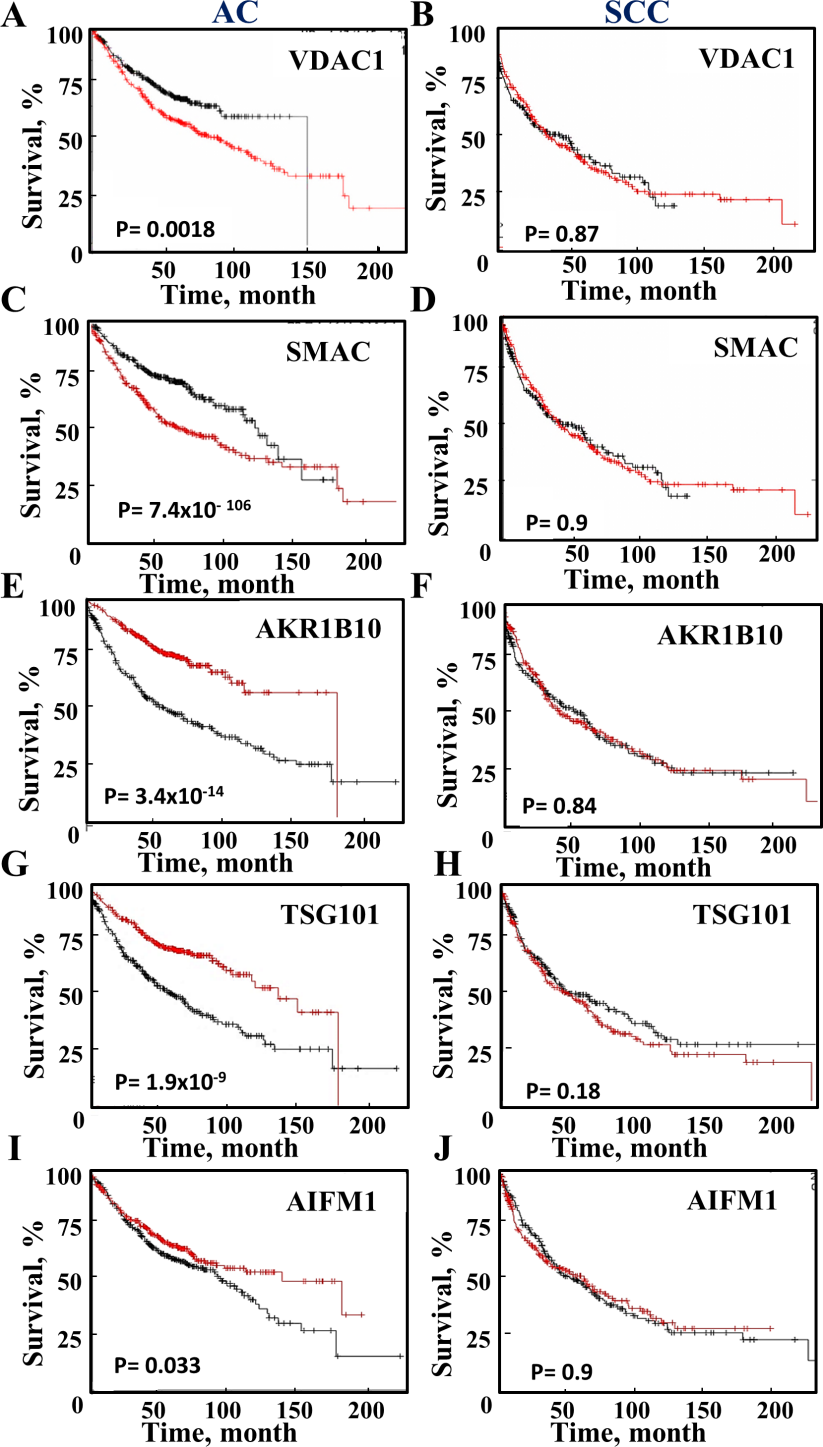
Survival of AC and SCC patients and the expression levels of selected proteins A Kaplan-Meier plot depicting patient survival rate with correlation to low (black) and high (red) expression of the proteins are presented for AC and SCC. The initial numbers of patients (for AC (n=720) and SCC (n=524) and the p-value are indicated in each graph. Data was obtained from http://KMplot.com.

To further test the prognostic value of the proteins proposed to distinguish between AC and SCC, survival analysis was performed on publicly available gene expression datasets of lung cancer patients (Figure 7). A Kaplan-Meier analysis assessing patient survival as a function of the relative indicated mRNA levels (high, red and low, black) in AC and SCC was performed. The results show that in AC patients, high levels of VDAC1, SMAC or HYOU1 are associated with low survival rates, while high levels of AKR1B10, AIF and TSG101 are associated with higher survival rates (Figure 7). In contrast, the expression levels of these proteins had no effect on SCC survival rates (Figure 7). A summary of the results with respect to survival and mRNA expression levels and the time for 50% death for AC and SCC patients is presented in Supplementary Table 8.

### SMAC is found in the nucleus in SCC and not in AC

**Figure 8:**
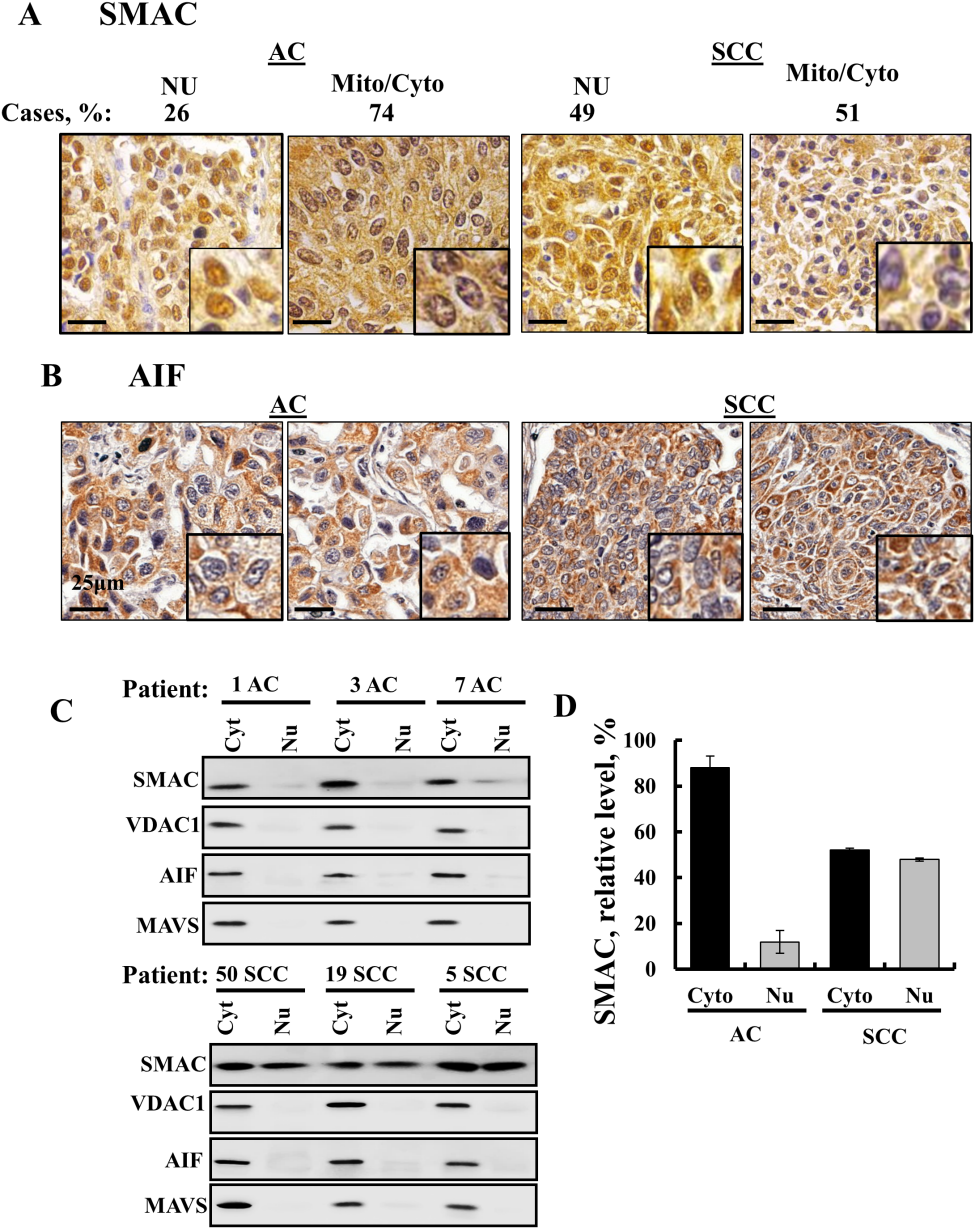
SMAC sub-cellular localization in lung cancer IHC staining of SMAC **(A)** and AIF **(B)** in human SCC and AC lung cancer in tissue array slides (US Biomax) with nuclear and cytosolic localization of SMAC shown. Case % represents the percentage of patient samples (72 AC and 72 SCC) that showed SMAC localization in the nucleus or cytosol and/ or mitochondria. Bar scale= 25 μm. **(C)** Nuclear extracts were prepared from AC and SCC lung cancer patient samples using a nuclear/cytosol fractionation kit (Biovision, Milpitas, CA), following the manufacturer's instructions. After centrifugation (16,000 g, 10 min), the supernatant (cytosolic fraction), and pellet (nuclear fraction) were re-suspended in the original volume and subjected to immunoblotting for SMAC, VDAC1 and AIF. **(D)** Quantitative analysis presenting the results as mean ± SEM (n = 3).

Interestingly, analysis of SMAC expression in a tissue array of lung cancer-derived samples revealed that although SMAC is a mitochondrial protein, high levels of the protein were found in the nucleus and cytosol of SCC but only to a lesser extent in AC (Figure 8A). No previous study has reported the presence of SMAC in the nucleus. The results further show that AIF, known to translocate to the nucleus upon apoptosis induction [16], is not present in the nucleus (Figure 8B).

Next, to further demonstrate the presence of SMAC in the nucleus, we analyzed the nuclear distribution of SMAC in AC and SCC lung cancer samples obtained from healthy and tumor tissues of the same lung after separating the nuclear and cytosolic fractions (Figure 8C, 8D). While in AC about 90% of SMAC was mitochondrial/cytosolic, in SCC about 50% was mitochondrial/cytosolic and 50% was found in the nuclear fraction (Figure 8D). In the nuclear fraction containing SMAC, three other mitochondrial proteins, VDAC1, MAVS and AIF, were not found (Figure 8C), indicating the specific nuclear localization of SMAC.

In summary, several biomarkers that potentially enable for distinguishing between AC and SCC, derived from published data, as confirmed here, or identified here for the first time, were selected based on being differentially expressed in SCC or AC (Supplementary Table 9).

## DISCUSSION

The identification of cancer biomarkers is a rapidly expanding field, with deep sequencing methods having become widely accepted as a means to detect and analyze cancer biomarkers. At the same time, other cancer-associated changes are not simply reflected as mutations in a gene but rather as increased or decreased expression or variations in post-translational modification of marker proteins, as reported in some cancers [17]. With this in mind, the study presented here identified alterations in the expression levels of metabolic, apoptotic and other proteins in NSCLC. These proteins can serve as potential means for a high sensitive platform that may allow better diagnosis of NSCLC and even early diagnosis, based on the secreted proteins AKR1B10, NPC2, GGH and AZGP1 (Supplementary Table 9). Most importantly, we identified proteins that allow for distinguishing between the AC and SCC sub-types, which is critical for accurate diagnosis and selection of treatment, particularly in unclear cases.

### Over-expression of metabolism-related proteins in NSCLC - potential biomarkers

The reprogramming of cellular metabolism is now a widely recognized hallmark of cancer [18]. Metabolic reprogramming require plasticity of the metabolic machinery to support the high proliferation rate of tumor cells and their unique metabolic demands. Both glycolysis and OXPHOS are reprogrammed, with the ratio depending on the prevalent normoxic or hypoxic environmental conditions and the capacity of a cell to express adequate levels of oncogenes and tumor suppressor gene products for growth [19]. Metabolic rewiring was also demonstrated in NSCLC, including enhanced production of glucose-derived TCA cycle intermediates [20].

Mitochondria in cancer cells maintain a membrane potential, oxidize respiratory substrates, and generate NADH and ATP, among other functions [21–24]. We found that the level of the mitochondrial gatekeeper protein, VDAC1, was substantially higher in different cancer types, in comparison to healthy tissues [25]. As such, we focused on its over-expression in NSCLC (Figures 2, 4A, and 6A, 6B). Previously, the VDAC1 gene expression level was reported to be increased in NSCLC, with this being associated with poor outcome [26]. As the main transporter of ions, Ca^2+^, ATP, and other metabolites across the outer mitochondrial membrane, VDAC1 over-expression could offer numerous advantages to highly energy-demanding cancer cells. Indeed, the requirement of VDAC1 for cancer development was demonstrated by silencing VDAC1 expression in cancer cells using specific si-RNA, resulting in marked inhibition of cancer cell proliferation both *in vitro* and *in vivo* [27, 28].

Other metabolism-related proteins that were also found here to be over-expressed in NSCLC include the glycolytic enzymes PGK1, LDHA, GAPDH, ENO1 and the OXPHOS protein ATP5B (Figure 3C) (Supplementary Table 5). Mitochondrial translocated PGK1 functions as a protein kinase, coordinating glycolysis and the TCA cycle in tumorigenesis [29] and acting in tumor angiogenesis as a disulphide reductase [30]. LDHA is over-expressed in several cancer types, including NSCLC [31]. GAPDH and ENO1 expression or polymorphism is associated with poor prognosis in NSCLC [32, 33]. Finally, ATP5B, a constituent of the F_1_F_0_ ATP synthase, was identified as a NSCLC tumor cellular membrane antigen [34].

Interestingly, network analysis (Figure 4) demonstrated that most of these proteins are connected by direct physical interactions or co-expression and some are encoded by a gene cluster that is regulated by epigenetic modifications. Most pronounced is the group of proteins associated with cell metabolic processes. Furthermore, this cluster includes genes encoding ATP5B, associated with oxidative phosphorylation (OXPHOS) and VDAC1, a gatekeeper of mitochondria, suggesting a coupling between OXPHOS and glycolysis, an important factor in cancer cell energy homeostasis (Warburg effect).

These results point to the significance of reprogrammed metabolism in NSCLC, as in other cancers [35], and that the listed proteins may serve as biomarkers.

### The pro-apoptotic proteins SMAC/Diablo and AIF are over-expressed in NSCLC – potential biomarkers

SMAC and AIF are normally located at the mitochondrial intermembrane space and released to the cytosol upon apoptotic signals [36]. Unexpectedlly, despite their pro-apoptotic function, SMAC and AIF were found to be over-expressed in NSCLC, as compared to healthy lung tissue (Figures 2, 5, 8). SMAC, as a pro-apoptotic protein, is released from mitochondria during apoptosis and counters the inhibitory activities of inhibitor of apoptosis proteins, IAPs, causing them to release their bound caspases [37]. SMAC was found to be over-expressed in some carcinomas [38–40] and sarcomas [41], yet showed reduced expression levels in other cancers [42]. This discrepancy between the increased SMAC expression level seen in many cancers and its pro-apoptotic activity [37] may result from another unidentified function of SMAC. Recently, we demonstrated the essential function of SMAC for cell and tumor growth in lung cancer [43].

AIF is also over-expressed in NSCLC (Figure 2). AIF, released to the cytosol upon apoptosis induction, translocates to the nucleus, where it triggers chromatin condensation and DNA degradation [16]. As a pro-apoptotic protein, it is not clear why AIF is over-expressed in cancer cells. AIF, however, has emerged as a protein critical for cell survival, as homozygous AIF knockout in mice is embryonically lethal [16]. The pro-survival activity of AIF was proposed to be related to oxidative phosphorylation, ROS detoxification, redox-sensing, mitochondrial morphology and cell cycle regulation [16]. Thus, AIF over-expression in some cancers may offer an advantage to cancer cells via these additional functions. AIF can thus serve as a NSCLC biomarker and as a new target for therapeutic approaches for the treatment of NSCLC.

### Proteins with modified expression in NSCLC as potential biomarkers

Proteomics (LC-HR MS/MS) analysis of healthy and NSCLC tissues from the same lung revealed several proteins that were highly expressed in the cancer, some of which were previously reported to be associated with other cancers and others reported as such for the first time here (Figures 1, 3, Supplementary Table 5). These proteins cover a spectrum of functional categories, such as tumor suppressors, protease inhibitors, structural proteins, RNA-binding factors, signaling of immune receptors, coordinators of mitochondrial peptide transmembrane transport or acting in amino acid, lipid or galactose metabolism or as protein kinases. Some of these are considered in more detail, below.

Rab11b protein was over-expressed (∼8000-fold) in the tumor tissues, yet was almost absent in the healthy lung tissues in all tested samples (Figure 3, Supplementary Table 5). The Rab11 family (Rab11a, Rab11b and Rab25) is associated with recycling endosomes, and only Rab25 was previously reported as being associated with cancer [44]. Vesicular trafficking in cancer has been suggested to regulate tumor invasion [45].

HYOU1, also known as HSP12A, GRP170 or ORP150, is over-expressed (∼60-fold) in lung cancer tissue (Figure 3, Supplementary Table 5). HYOU1 is proposed to play an important role in protein folding and secretion in the ER, and contributes to cytoprotection in hypoxia-induced cellular perturbation [46]. HYOU1 was shown to be up-regulated in breast and nasopharyngeal carcinomas, and was associated with tumor invasiveness and poor prognosis (Supplementary Table 5).

EGFR and MEK1 were found to be over-expressed in the tumor, as compared to healthy lung tissues (Supplementary Table 5). Hyper-activation of the EGFR-Ras-MAPK pathway, where mutant proteins are involved, is the most common alteration in lung cancer [7, 47, 48]. Thus, many of these proteins may serve as NSCLC biomarkers.

### Biomarkers for SCC and AC diagnosis- SMAC in the nucleus signature for SCC

The two main sub-types of NSCLC, AC and SCC, show differences in genome mutations, and in the epigenome, transcriptome, and proteome [7]. Thyroid transcription factor-1 (TTF-1) is currently used in the clinic to distinguish between AC and SCC, [49]. Nevertheless, it is still a challenge distinguishing between these two NSCLC sub-types [50]. Precise diagnosis is essential for selecting the appropriate treatment and thus increasing patient life expectancy.

Here, we present newly identified proteins that allow for distinguishing between AC and SCC and also confirm several previously reported proteins (Supplementary Tables 6, 9). USP14 and AKR1B10 were found to be over-expressed in SCC (Figure 5B, 5C). AKR1B10 has been previously reported as a potential diagnostic marker specific to smokers' NSCLCs, while USP14 was reported to be over-expressed in various types of cancer, including NSCLC (Supplementary Tables 5). Other proteins found to be over-expressed in SCC are TTL12 and HAT1, previously reported to be associated with prostate cancer or lymphoma and esophageal squamous cell carcinoma progression, respectively (Supplementary Tables 6 and 9). LRRFIP2, WDR82 and ACOT1 were not identified previously as possible biomarkers for any type of cancer (Figure 5B, 5C). In contrast, TSG101, involved in lung cancer cell proliferation, RAB34, HYOU1 and ACOT1 showed higher expression in AC, as compared to their levels in SCC (Figure 5, Supplementary Tables 6 and 9). Finally, the expression levels of these proteins affected AC patient survival but had no effect on SCC survival (Figure 7, Supplementary Table 8).

The proteins selected based on their differential expression levels in AC and SCC, as revealed by LC-HR MS/MS (Figure 5B, 5C), also differed at the RNA level in SCC and AC (Figure 6A, 6B). Further analysis of RNAseq UCSC XENA data, selecting for genes encoding proteins showing differential expression levels in AC and SCC (LC-HR MS/MS data), identified additional biomarkers. mRNA levels encoding for proteins associated with a variety of functions were changed in AC and SCC (3-60-fold) (Figure 6C, 6D). Of interest are proteins previously proposed as biomarkers for SCC, such as TP63 and Ck5, Ck13, Ck14, Ck17, CSTA and PFN2 (Figure 6C, 6D and Supplementary Table 7). The over-expression of the cytokeratin genes Ck5, Ck13, Ck14 and Ck17 in SCC is in agreement with such cancer originating from squamous epithelium cells and the physiological function of these proteins (Supplementary Table 7). Thus, these four cytokeratins may allow for better and precise diagnosis of SCC. While AKR7A3 and ACAD8 were identified here for the first time as being over-expressed in AC (2-6-fold), relative to their expression levels in SCC (Figure 6C, 6D), genes such as NPC2 (Niemann-Pick disease, type C2) [51], encoding a secreted protein, and ARRB1, were previously reported as biomarkers for lung AC and confirmed here (Supplementary Table 9).

Another interesting group of genes that are highly expressed in AC, relative to SCC, are those associated with fatty acid/lipid metabolism and transport, such as AZGP1 (zinc-alpha2-glycoprotein) [52], a secreted protein that stimulates lipid degradation in adipocytes and causes the extensive fat losses associated with some advanced cancers [53]. NPC2 facilitates intracellular cholesterol transport [54], ACOT1 (acyl-CoA thioesterase 1) is a secreted protein thatis a regulator of peroxisomal lipid metabolism [55], while ACAD8 (isobutyryl-CoA dehydrogenase) is a mitochondrial protein catalyzing the dehydrogenation of acyl-CoA derivatives in the metabolism of fatty acids or branched- chain amino acids, such as valine [56]. In this respect, AC mostly originates from alveolar type 2 (AT2) cells, with lipid metabolism systems being part of surfactant production associated with these cells.

Most interestingly is the cellular localization of SMAC, being found not only in mitochondria but also in the nucleus, specifically in the nuclei of SCC samples (Figure 8). Thus, the presence of SMAC in the nucleus may be a clear signature for SCC.

Collectively, based on expression level changes (fold change) and specific expression in AC or SCC of protein/mRNA identified here for the first time, or in previous reports and confirmed here, we propose a list of proteins differentially expressed in SCC and AC, of which four are secreted proteins (Supplementary Table 9), that can be used to clearly distinguish between SCC or AC. This is most important for guiding the appropriate treatment for these two NSCLC sub-types.

In summary, we have identified several proteins whose expression levels are highly increased in lung cancer patients. Moreover, some of these biomarkers can be used as profiling platforms to enable one to distinguish between AC and SCC. The use of these molecules may facilitate accurate diagnosis and prognostic prediction and could contribute to individualized lung cancer treatment. Finally, the search for drugs that target these biomarkers may lead to new treatments for lung cancer patients.

## MATERIALS AND METHODS

### Materials

Phenylmethylsulfonyl fluoride (PMSF), propidium iodide (PI), and trypan blue were purchased from Sigma (St. Louis, MO). Dulbecco's modified Eagle's medium (DMEM) and the supplements fetal calf serum, L-glutamine and penicillin-streptomycin were purchased from Biological Industries (Beit Haemek, Israel). Primary antibodies, their sources, and the dilutions used are detailed in Supplementary Table 1. Horseradish peroxidase (HRP)-conjugated anti-mouse, anti-rabbit and anti-goat antibodies were from KPL (Gaithersburg, MD). 3,3-diaminobenzidine (DAB) was obtained from ImmPact-DAB (Burlingame, CA). Primary antibodies used in immunoblotting and immunohistochemistry (IHC), as well as their dilutions, are listed in Supplementary Table 2.

### Patients

All the investigations represented in this study were conducted after informed consent was obtained and in accordance with an institutional review board protocol approved by the Ethics Committee of Soroka University Medical Center. All human tissues were collected with the understanding and written consent of each subject, and the study methodologies conformed to the standards set by the Declaration of Helsinki.

NSCLC specimens were obtained from 2010 to 2016 from 46 patients who underwent lung resection without any treatment at the time of surgery. The main clinical and pathologic variables of the patients are provided in Supplementary Table 3.

Fresh paired healthy and cancer tissue specimens were obtained from the same lung cancer patients undergoing either pneumonectomy or pulmonary lobectomy to remove tumors tissue and were immediately frozen in liquid nitrogen and maintained at −80°C until analysis by immunoblotting or q-PCR. Proteins were extracted from the tissue samples as described below. Cancer and healthy lung tissue surrounding the tumor were validated by hospital pathologists.

### Biomax tissue arrays

Cancer tissue microarrays were purchased from Biomax US (**US Biomax)**. These included arrays for lung cancer (LC807) containing lung healthy tissues (n=10) and various lung cancer types in different stages, including AC (n=21), adenosquamous carcinoma (n=1), SCC (n=31), bronchioloalveolar carcinoma (BAC; n=6), small cell carcinoma (n=6) and large cell carcinoma (n=5). A second tissue array (BC041115c) contained healthy lung tissue (n=10), AC (n=51) and SCC (n=41) tissue samples.

### Immunohistochemistry (IHC)

Immunohistochemical staining was performed on formalin-fixed and paraffin-embedded tissue microarray slides (US Biomax). The slides were subjected to deparaffinized antigen retrieval, IHC using the antibodies listed in Supplementary Table 2 and image photography, as described in Supplementary Data.

### Protein extraction from lung tissue

To extract proteins for immunoblotting, lung healthy and tumor tissues were solubilized in a lysis buffer (50 mM Tris-HCl, pH 7.5, 150 mM NaCl, 1 mM EDTA, 1.5 mM MgCl_2_, 10% glycerol, 1% Triton X-100, a protease inhibitor cocktail (Calbiochem)), followed by sonication and centrifugation (10 min, 600 g). The protein concentration of each lysate was determined using a Lowry assay. Samples were stored at −80°C until analysis by gel electrophoresis and immunoblotting, as described in Supplementary Data.

To extract proteins for LC-HR MS/MS, lung healthy and tumor tissues were solubilized in a lysis buffer (100 mM Tris-HCl, pH 8.0, 5 mM DTT 4% SDS and a protease inhibitor cocktail (Calbiochem;100 μl/10 mg)), followed by homogenization, incubation for 3 min at 95°C and centrifugation (10 min, 15,000 g). The protein concentration of each lysate was determined using a Lowry assay. Samples were stored at −80°C until MS/MS analysis, as described in Supplementary Data.

### Gel electrophoresis, immunoblotting and q-PCR

Samples were subjected to SDS-PAGE and immunostaining with various primary antibodies and to quantitative analysis by q-PCR using specific primers (Supplementary Table 4), as described in the Supplementary Data.

### LC-HR MS/MS analysis

Healthy and cancerous lung tissue samples were analyzed from each of nine AC patients and in additional experiment from 5 AC and 5 SCC patients. The samples were subjected to in-solution tryptic digestion and LC-HR MS/MS. Data analysis was carried out as described in the Supplementary Data.

### RNAseq gene expression profiling

Data for the gene expression profile and for the heat map for healthy and tumor lung samples of lung cancer patients were obtained from XENA, TCGA [RNAseq using ployA+ Illumina HiSeq] (version 2016-08-16, TCGA hub) (http://xena.ucsc.edu), with the unit being pan-cancer-normalized (n=1,129). Linear fold change and statistical analysis were performed using a t-test.

### Statistics and bioinformatics analysis

All descriptive statistics for data analysis were computed using the SPSS statistical package, version 17.0. Means ± SEM of results obtained from the indicated independent experiments are presented. The level of significance of differences between the control (healthy) and experimental (cancer) groups was determined by a non-parametric Mann-Whitney U test. A difference was considered statistically significant when the p-value was deemed <0.05 (^*^), <0.01 (^**^) or <0.001 (^***^).

LC-HR-MS/MS data were imported into Partek Genomics Suite software (Partek, St. Louis, MO) and differences between expression levels of the proteins in the different groups were calculated using a t-test. Functional enrichment analysis of differentially expressed proteins was performed using the DAVID Gene Ontology (GO) bioinformatics resources, v6.7 [57].

## SUPPLEMENTARY MATERIALS FIGURES AND TABLES





## Abbreviations

AC: adenocarcinoma
AIF: apoptosis-inducing factor
CLL: chronic lymphocytic leukemia
HK-I: hexokinase-I
MAVS: mitochondrial antiviral-signaling
SCC: squamous cell carcinoma
SMAC/Diablo: second mitochondria-derived activator of caspase/direct inhibitor of apoptosis-binding protein with low pI
VDAC1: voltage-dependent anion channel 1
