# Supplementary material for: A molecular signature of lung cancer: potential biomarkers for adenocarcinoma and squamous cell carcinoma
Source: Oncotarget. 2017 Nov 6;8(62):105492–509. doi: 10.18632/oncotarget.22298 (PMC5739654; doi:10.18632/oncotarget.22298)
Supplement: Supplementary file 1 [file oncotarget-08-105492-s001.pdf]

A molecular signature of lung cancer: potential biomarkers for adenocarcinoma and squamous cell carcinoma

SUPPLEMENTARY MATERIALS

Materials and methods

Gel electrophoresis and immunoblotting

Samples (10-40 µg of protein) were subjected to SDS-PAGE. Gels were stained with Coommassie Brilliant Blue or electro-transferred onto nitrocellulose membranes for immunostaining. Membranes containing the transferred proteins were blocked with 5% non-fat dry milk and 0.1% Tween-20 in Tris-buffered saline (TBS) and incubated overnight at 4°C with the different primary antibodies (sources and dilutions as detailed in Supplementary Table 2), followed by incubation with the appropriate HRP-conjugated secondary antibodies for 1 h. Enhanced chemiluminescence (Biological Industries) was used for detection of HRP activity. Band intensities were analyzed using FUSION-FX (Vilber Lourmat, France) and the values were normalized to the intensities of the appropriate α-actin signal that served as a loading control.

RNA isolation and q-PCR

Total RNA was isolated from healthy and tumor lung samples using an RNeasy mini kit (Qiagen) according to the manufacturer's instructions. Total RNA quality was analyzed using the Agilent RNA 6000 nano kit. q-PCR was performed using specific primers (KiCqStart Primers; Sigma Aldrich) in triplicate, using Power SYBER green master mix (Applied Biosystems, Foster City, CA). Levels of target genes were normalized relative to β-actin mRNA levels. Samples were amplified by a 7300 Real Time PCR System (Applied Biosystems) for 40 cycles using the following PCR parameters: 95°C for 15 seconds, 60°C for 1 minute, and 72°C for 1 minute. The copy numbers for each sample were calculated by the CT-based calibrated standard curve method. The mean fold changes (± SEM) of the three replicates were calculated. Genes examined and primers used are listed in Supplementary Table 4.

LC-HR MS/MS analysis

Samples were subjected to in-solution tryptic digestion as follows. Proteins were first reduced by incubation with 5 mM DTT for 30 min at 60°C, followed by alkylation with 10 mM iodoacetamide in the dark for 30 min at 21°C. Proteins were then subjected to digestion with trypsin (Promega, Madison, WI) at a 1:50 trypsin:protein ratio for 16 h at 37°C. Following digestion, detergents were cleared from the samples using commercial detergent removal columns (Pierce, Rockford, IL), and desalted using solid-phase extraction columns (Oasis HLB, Waters, Milford, MA). Digestions were stopped by addition of trifluroacetic acid (1%). The samples were stored at -80°C until LC-HR MS/MS analysis.

For LC-HR MS/MS, ULC/MS grade solvents were used for all chromatographic steps. Each sample was separated using split-less nano-ultra performance liquid chromatography columns (10 kpsi nanoAcquity; Waters). The mobile phase was (A) H_2O and 0.1% formic acid, and (B) acetonitrile and 0.1% formic acid. Desalting of the samples was performed online using a reverse-phase C18 trapping column (180 µm internal diameter, 20 mm length, 5 µm particle size; Waters). The peptides were then separated using a T3 HSS nano-column (75 µm internal diameter, 250 mm length, 1.8 µm particle size; Waters) at 0.3 µL/min. Peptides were eluted from the column into the mass spectrometer using the following gradient: 4% to 35% (B) for 150 min, 35% to 90% (B) for 5 min, maintained at 90% for 5 min and then back to initial conditions. The nano-UPLC was coupled online through a nano-ESI emitter (10 µm tip; New Objective, Woburn, MA) to a quadrupole Orbitrap mass spectrometer (Q Executive, Thermo Scientific) using a FlexIon nanospray apparatus (Proxeon). Data was acquired in the DDA mode, using a Top12 method [1]. Raw data was imported into Expressionist software (Genedata) [2, 3], The software was used for retention time alignment and peak detection of precursor peptide intensities. A master peak list was generated from all MS/MS events and sent for database searching using Mascot v2.4 (Matrix Sciences). Data was searched against a database containing forward and reverse human protein sequences from UniprotKB/SwissProt, and 125 common laboratory contaminants, totaling 20,304 entries. Fixed modification was set to carbamidomethylation of cysteines, while variable modification was set to oxidation of methionines. Search results were then imported back to Expressionist for annotation of detected peaks. Identifications were filtered such that the global false discovery rate was a maximum of 1%. Protein abundance was calculated based on the three most abundant peptides [4].

Proteins with less than 2 unique peptides were excluded from further analysis.

Samples from 9 patients were analyzed, with healthy and cancerous lung tissues being taken from the

same patient lung. Proteins for which at least two unique peptides were identified were used for further analysis.

Immunohistochemistry (IHC) on tissue microarray (TMA) slides

Immunohistochemical staining was performed on formalin-fixed and paraffin-embedded tissue microarray slides obtained from Biomax US. The sections were deparaffinized using xylene and a graded ethanol series. Endogenous peroxidase activity was blocked by incubating the sections in 3% H_2O_2 for 10 minutes. Antigen retrieval was performed in 0.01M citrate buffer (pH 6.0) at 95°C-98°C for 20 min. After washing sections in PBS (pH 7.4), non-specific antibody binding was reduced by incubating the sections in 10% normal goat serum for 2 h. After decanting excess serum, sections were incubated overnight at 4°C with primary antibodies (Supplementary Table 2). After washing with PBS, the sections were incubated for 2 h with the appropriate secondary antibodies conjugated to horseradish peroxidase (Supplementary Table 2). Sections were washed three times in PBS and subsequently, the peroxidase-catalyzed reaction was visualized by incubating with 0.02% DAB. After rinsing in water, the sections were counterstained with hematoxylin, and mounted with Vectashield mounting medium (Vector Laboratories, Burlingame, CA). Finally, the sections were observed under a microscope (DM2500, Leica) and images were taken at the indicated magnification with the same light intensity and exposure time. Controls were carried out with the same protocols but omitting the primary antibodies.

REFERENCES

1. Kelstrup CD, Young C, Lavallee R, Nielsen ML, Olsen JV. Optimized fast and sensitive acquisition methods for shotgun proteomics on a quadrupole orbitrap mass spectrometer. J Proteome Res. 2012; 11: 3487-97. https://doi.org/10.1021/pr3000249.
2. Ueda K, Saichi N, Takami S, Kang D, Toyama A, Daigo Y, Ishikawa N, Kohno N, Tamura K, Shuin T, Nakayama M, Sato TA, Nakamura Y, et al. A comprehensive peptidome profiling technology for the identification of early detection biomarkers for lung adenocarcinoma. PLoS One. 2011; 6: e18567. https://doi.org/10.1371/journal.pone.0018567.
3. Guryca V, Lamerz J, Ducret A, Cutler P. Qualitative improvement and quantitative assessment of N-terminomics. Proteomics. 2012; 12: 1207-16. https://doi.org/10.1002/pmic.201100430.
4. D'Arena G, Laurenti L, Capalbo S, D'Arco AM, De Filippi R, Marcacci G, Di Renzo N, Storti S, Califano C, Vigliotti ML, Tarnani M, Ferrara F, Pinto A. Rituximab therapy for chronic lymphocytic leukemia-associated autoimmune hemolytic anemia. Am J Hematol. 2006; 81: 598-602. https://doi.org/10.1002/ajh.20665.
5. Campbell JD, Alexandrov A, Kim J, Wala J, Berger AH, Pedamallu CS, Shukla SA, Guo G, Brooks AN, Murray BA, Imielinski M, Hu X, Ling S, et al. Distinct patterns of somatic genome alterations in lung adenocarcinomas and squamous cell carcinomas. Nat Genet. 2016; 48: 607-16. https://doi.org/10.1038/ng.3564
6. Paez JG, Janne PA, Lee JC, Tracy S, Greulich H, Gabriel S, Herman P, Kaye FJ, Lindeman N, Boggon TJ, Naoki K, Sasaki H, Fujii Y, et al. EGFR mutations in lung cancer: correlation with clinical response to gefitinib therapy. Science. 2004; 304: 1497-500. https://doi.org/10.1126/science.1099314
7. Mitsudomi T, Yatabe Y. Mutations of the epidermal growth factor receptor gene and related genes as determinants of epidermal growth factor receptor tyrosine kinase inhibitors sensitivity in lung cancer. Cancer Sci. 2007; 98: 1817-24. doi: CAS607 [pii]10.1111/j.1349-7006.2007.00607.x.
8. Saito M, Suzuki H, Kono K, Takenoshita S, Kohno T. Treatment of lung adenocarcinoma by molecular-targeted therapy and immunotherapy. Surgery Today. 2017: 1-8. https://doi.org/10.1007/s00595-017-1497-7.
9. Plönes T, Engel-Riedel W, Stoelben E, Limmroth C, Schildgen O, Schildgen V. Molecular Pathology and Personalized Medicine: The Dawn of a New Era in Companion Diagnostics—Practical Considerations about Companion Diagnostics for Non-Small-Cell-Lung-Cancer. Journal of Personalized Medicine. 2016; 6: 3. https://doi.org/10.3390/jpm6010003.
10. Li T, Maus MK, Desai SJ, Beckett LA, Stephens C, Huang E, Hsiang J, Zeger G, Danenberg KD, Astrow SH, Gandara DR. Large-scale screening and molecular characterization of EML4-ALK fusion variants in archival non-small-cell lung cancer tumor specimens using quantitative reverse transcription polymerase chain reaction assays. J Thorac Oncol. 2014; 9: 18-25. https://doi.org/10.1097/JTO.035491R1035491R10030.
11. Cao B, Wei P, Liu Z, Bi R, Lu Y, Zhang L, Zhang J, Yang Y, Shen C, Du X, Zhou X. Detection of lung adenocarcinoma with ROS1 rearrangement by IHC, FISH, and RT-PCR and analysis of its clinicopathologic features. OncoTargets and therapy. 2016; 9: 131-8. https://doi.org/10.2147/OTT.S94997.
12. Lee M-S, Kim RN, I H, Oh D-Y, Song J-Y, Noh K-W, Kim Y-J, Yang JW, Lira ME, Lee CH, Lee MK, Kim YD, Mao M, et al. Identification of a novel partner gene, KIAA1217, fused to RET: Functional characterization and inhibitor sensitivity of two isoforms in lung adenocarcinoma. Oncotarget. 2016; 7: 36101-14. https://doi.org/10.18632/oncotarget.9137.

13. Benedettini E, Sholl LM, Peyton M, Reilly J, Ware C, Davis L, Vena N, Bailey D, Yeap BY, Fiorentino M, Ligon AH, Pan BS, Richon V, et al. Met activation in non-small cell lung cancer is associated with de novo resistance to EGFR inhibitors and the development of brain metastasis. Am J Pathol. 2010; 177: 415-23. https://doi.org/10.2353/ajpath.2010.090863.
14. Nakamura Y, Niki T, Goto A, Morikawa T, Miyazawa K, Nakajima J, Fukayama M. c-Met activation in lung adenocarcinoma tissues: an immunohistochemical analysis. Cancer Sci. 2007; 98: 1006-13. https://doi.org/10.1111/j.1349-7006.2007.00493.x.
15. Rachwal WJ, Bongiorno PF, Orringer MB, Whyte RI, Ethier SP, Beer DG. Expression and activation of erbB-2 and epidermal growth factor receptor in lung adenocarcinomas. British Journal of Cancer. 1995; 72: 56-64.
16. Vargas AJ, Harris CC. Biomarker development in the precision medicine era: lung cancer as a case study. Nature Reviews Cancer. 2016; 16: 525-37.
17. Campbell JD, Alexandrov A, Kim J, Wala J, Berger AH, Pedamallu CS, Shukla SA, Guo G, Brooks AN, Murray BA, Imielinski M, Hu X, Ling S, et al. Distinct patterns of somatic genome alterations in lung adenocarcinomas and squamous cell carcinomas. Nat Genet. 2016; 48: 607-16. https://doi.org/10.1038/ng.3564.
18. Ao MH, Zhang H, Sakowski L, Sharma R, Illei PB, Gabrielson E, Askin F, Li QK. The utility of a novel triple marker (combination of TTF1, napsin A, and p40) in the subclassification of non-small cell lung cancer. Hum Pathol. 2014; 45: 926-34. https://doi.org/10.1016/j.humpath.2014.01.005 S0046-8177(14)00029-X [pii].
19. Vogt AP, Cohen C, Siddiqui MT. p40 (DeltaNp63) is more specific than p63 and cytokeratin 5 in identifying squamous cell carcinoma of bronchopulmonary origin: a review and comparative analysis. Diagn Cytopathol. 2014; 42: 453-8. https://doi.org/10.1002/dc.23045.
20. Kim MJ, Shin HC, Shin KC, Ro JY. Best immunohistochemical panel in distinguishing adenocarcinoma from squamous cell carcinoma of lung: tissue microarray assay in resected lung cancer specimens. Ann Diagn Pathol. 2013; 17: 85-90. https://doi.org/10.1016/j.anndiagpath.2012.07.006 S1092-9134(12)00103-7 [pii].
21. Ogawa H, Yonezawa S, Maruyama I, Matsushita Y, Tezuka Y, Toyoyama H, Yanagi M, Matsumoto H, Nishijima H, Shimotakahara T, Aikou T, Sato E. Expression of thrombomodulin in squamous cell carcinoma of the lung: its relationship to lymph node metastasis and prognosis of the patients. Cancer Lett. 2000; 149: 95-103.
22. Tolnay E, Wiethege T, Muller KM. Expression and localization of thrombomodulin in preneoplastic bronchial lesions and in lung cancer. Virchows Arch. 1997; 430: 209-12.
23. Gez S, Crossett B, Christopherson RI. Differentially expressed cytosolic proteins in human leukemia and lymphoma cell lines correlate with lineages and functions. Biochim Biophys Acta. 2007; 1774: 1173-83. https://doi.org/10.1016/j.bbapap.2007.06.011.
24. Nagpal JK, Dasgupta S, Jadallah S, Chae YK, Ratovitski EA, Toubaji A, Netto GJ, Eagle T, Nissan A, Sidransky D, Trink B. Profiling the expression pattern of GPI transamidase complex subunits in human cancer. Mod Pathol. 2008; 21: 979-91. https://doi.org/10.1038/modpathol.2008.76.
25. Lombardo Y, Filipovic A, Molyneux G, Periyasamy M, Giamas G, Hu Y, Trivedi PS, Wang J, Yague E, Michel L, Coombes RC. Nicastrin regulates breast cancer stem cell properties and tumor growth *in vitro* and *in vivo*. Proc Natl Acad Sci U S A. 2012; 109: 16558-63. https://doi.org/10.1073/pnas.1206268109.
26. Fukuda M, Ishii A, Yasutomo Y, Shimada N, Ishikawa N, Hanai N, Nagata N, Irimura T, Nicolson GL, Kimura N. Decreased expression of nucleoside diphosphate kinase alpha isoform, an nm23-H2 gene homolog, is associated with metastatic potential of rat mammary-adenocarcinoma cells. Int J Cancer. 1996; 65: 531-7. https://doi.org/10.1002/(SICI)1097-0215(19960208)65:4<531::AID-IJC23>3.0.CO;2-B.
27. Russell RL, Pedersen AN, Kantor J, Geisinger K, Long R, Zbieranski N, Townsend A, Shelton B, Brunner N, Kute TE. Relationship of nm23 to proteolytic factors, proliferation and motility in breast cancer tissues and cell lines. Br J Cancer. 1998; 78: 710-7.
28. Edick MJ, Cheng C, Yang W, Cheok M, Wilkinson MR, Pei D, Evans WE, Kun LE, Pui CH, Relling MV. Lymphoid gene expression as a predictor of risk of secondary brain tumors. Genes Chromosomes Cancer. 2005; 42: 107-16. https://doi.org/10.1002/gcc.20121.
29. Miao P, Sheng S, Sun X, Liu J, Huang G. Lactate dehydrogenase A in cancer: a promising target for diagnosis and therapy. IUBMB Life. 2013; 65: 904-10. https://doi.org/10.1002/iub.1216.
30. Cerutti JM, Latini FR, Nakabashi C, Delcelo R, Andrade VP, Amadei MJ, Maciel RM, Hojaij FC, Hollis D, Shoemaker J, Riggins GJ. Diagnosis of suspicious thyroid nodules using four protein biomarkers. Clin Cancer Res. 2006; 12: 3311-8. https://doi.org/10.1158/1078-0432.CCR-05-2226.
31. Sudo H, Tsuji AB, Sugyo A, Kohda M, Sogawa C, Yoshida C, Harada YN, Hino O, Saga T. Knockdown of COPA, identified by loss-of-function screen, induces apoptosis and suppresses tumor growth in mesothelioma mouse model.

Genomics. 2010; 95: 210-6. https://doi.org/10.1016/j.ygeno.2010.02.002.

32. Li Y, Gao Y, Xu Y, Sun X, Song X, Ma H, Yang M. si-RNA-mediated knockdown of PDLIM5 suppresses gastric cancer cell proliferation *in vitro*. Chem Biol Drug Des. 2015; 85: 447-53. https://doi.org/10.1111/cbdd.12428.
33. Cha JD, Kim HJ, Cha IH. Genetic alterations in oral squamous cell carcinoma progression detected by combining array-based comparative genomic hybridization and multiplex ligation-dependent probe amplification. Oral Surg Oral Med Oral Pathol Oral Radiol Endod. 2011; 111: 594-607. https://doi.org/10.1016/j.tripleo.2010.11.020.
34. Symes AJ, Eilertsen M, Millar M, Nariculam J, Freeman A, Notara M, Feneley MR, Patel HR, Masters JR, Ahmed A. Quantitative analysis of BTF3, HINT1, NDRG1 and ODC1 protein over-expression in human prostate cancer tissue. PLoS One. 2013; 8: e84295. https://doi.org/10.1371/journal.pone.0084295.
35. Oue N, Naito Y, Hayashi T, Takigahira M, Kawano-Nagatsuma A, Sentani K, Sakamoto N, Zarni Oo H, Uraoka N, Yanagihara K, Ochiai A, Sasaki H, Yasui W. Signal peptidase complex 18, encoded by SEC11A, contributes to progression via TGF-alpha secretion in gastric cancer. Oncogene. 2014; 33: 3918-26. https://doi.org/10.1038/onc.2013.364.
36. Stary S, Vinatzer U, Mullauer L, Raderer M, Birner P, Streubel B. t(11;14)(q23;q32) involving IGH and DDX6 in nodal marginal zone lymphoma. Genes Chromosomes Cancer. 2013; 52: 33-43. https://doi.org/10.1002/gcc.22004.
37. Sun S, Liang X, Zhang X, Liu T, Shi Q, Song Y, Jiang Y, Wu H, Jiang Y, Lu X, Pang D. Phosphoglycerate kinase-1 is a predictor of poor survival and a novel prognostic biomarker of chemoresistance to paclitaxel treatment in breast cancer. Br J Cancer. 2015; 112: 1332-9. https://doi.org/10.1038/bjc.2015.114.
38. Avdulov S, Herrera J, Smith K, Peterson M, Gomez-Garcia JR, Beadnell TC, Schwertfeger KL, Benyumov AO, Manivel JC, Li S, Bielinsky AK, Yee D, Bitterman PB, et al. eIF4E threshold levels differ in governing normal and neoplastic expansion of mammary stem and luminal progenitor cells. Cancer Res. 2015; 75: 687-97. https://doi.org/10.1158/0008-5472.CAN-14-2571.
39. Wang JW, Peng SY, Li JT, Wang Y, Zhang ZP, Cheng Y, Cheng DQ, Weng WH, Wu XS, Fei XZ, Quan ZW, Li JY, Li SG, et al. Identification of metastasis-associated proteins involved in gallbladder carcinoma metastasis by proteomic analysis and functional exploration of chloride intracellular channel 1. Cancer Lett. 2009; 281: 71-81. https://doi.org/10.1016/j.canlet.2009.02.020.
40. Sun ZL, Zhu Y, Wang FQ, Chen R, Peng T, Fan ZN, Xu ZK, Miao Y. Serum proteomic-based analysis of pancreatic carcinoma for the identification of potential cancer biomarkers. Biochim Biophys Acta. 2007; 1774: 764-71. https://doi.org/10.1016/j.bbapap.2007.04.001.
41. Lai MD, Xu J. Ribosomal proteins and colorectal cancer. Curr Genomics. 2007; 8: 43-9.
42. Yoshihara K, Tajima A, Adachi S, Quan J, Sekine M, Kase H, Yahata T, Inoue I, Tanaka K. Germline copy number variations in BRCA1-associated ovarian cancer patients. Genes Chromosomes Cancer. 2011; 50: 167-77. https://doi.org/10.1002/gcc.20841.
43. Takahashi H, Nishimura J, Kagawa Y, Kano Y, Takahashi Y, Wu X, Hiraki M, Hamabe A, Konno M, Haraguchi N, Takemasa I, Mizushima T, Ishii M, et al. Significance of Polypyrimidine Tract-Binding Protein 1 Expression in Colorectal Cancer. Mol Cancer Ther. 2015; 14: 1705-16. https://doi.org/10.1158/1535-7163.MCT-14-0142.
44. Calabretta S, Bielli P, Passacantilli I, Pilozzi E, Fendrich V, Capurso G, Fave GD, Sette C. Modulation of PKM alternative splicing by PTBP1 promotes gemcitabine resistance in pancreatic cancer cells. Oncogene. 2016; 35: 2031-9. https://doi.org/10.1038/onc.2015.270.
45. He X, Arslan AD, Ho TT, Yuan C, Stampfer MR, Beck WT. Involvement of polypyrimidine tract-binding protein (PTBP1) in maintaining breast cancer cell growth and malignant properties. Oncogenesis. 2014; 3: e84. https://doi.org/10.1038/oncsis.2013.47.
46. Gu J, Xu FK, Zhao GY, Lu CL, Lin ZW, Ding JY, Ge D. Capn4 promotes non-small cell lung cancer progression via upregulation of matrix metalloproteinase 2. Med Oncol. 2015; 32: 51. https://doi.org/10.1007/s12032-015-0500-7.
47. Zheng PC, Chen X, Zhu HW, Zheng W, Mao LH, Lin C, Liu JN, Zheng M. Capn4 is a marker of poor clinical outcomes and promotes nasopharyngeal carcinoma metastasis via nuclear factor-kappaB-induced matrix metalloproteinase 2 expression. Cancer Sci. 2014; 105: 630-8. https://doi.org/10.1111/cas.12416.
48. Kohnz RA, Mulvihill MM, Chang JW, Hsu KL, Sorrentino A, Cravatt BF, Bandyopadhyay S, Goga A, Nomura DK. Activity-Based Protein Profiling of Oncogene-Driven Changes in Metabolism Reveals Broad Dysregulation of PAFAH1B2 and 1B3 in Cancer. ACS Chem Biol. 2015; 10: 1624-30. https://doi.org/10.1021/acschembio.5b00053.
49. Bazzocco S, Dopeso H, Carton-Garcia F, Macaya I, Andretta E, Chionh F, Rodrigues P, Garrido M, Alazzouzi H, Nieto R, Sanchez A, Schwartz S, Jr., Bilic J, et al. Highly Expressed Genes in Rapidly Proliferating Tumor Cells as New Targets for Colorectal Cancer Treatment. Clin Cancer Res. 2015; 21: 3695-704. https://doi.org/10.1158/1078-0432.CCR-14-2457.
50. Cao XX, Xu JD, Liu XL, Xu JW, Wang WJ, Li QQ, Chen Q, Xu ZD, Liu XP. RACK1: A superior independent predictor for poor clinical outcome in breast cancer. Int J Cancer. 2010; 127: 1172-9. https://doi.org/10.1002/ijc.25120.

51. Guo Y, Wang W, Wang J, Feng J, Wang Q, Jin J, Lv M, Li X, Li Y, Ma Y, Shen B, Zhang J. Receptor for activated C kinase 1 promotes hepatocellular carcinoma growth by enhancing mitogen-activated protein kinase kinase 7 activity. Hepatology. 2013; 57: 140-51. https://doi.org/10.1002/hep.25978.
52. Sancak Y, Markhard AL, Kitami T, Kovács-Bogdán E, Kamer KJ, Udeshi ND, Carr SA, Chaudhuri D, Clapham DE, Li AA, Calvo SE, Goldberger O, Mootha VK. EMRE Is an Essential Component of the Mitochondrial Calcium Uniporter Complex. Science. 2013; 342: 1379-82.
53. Peng R, Jiang B, Ma J, Ma Z, Wan X, Liu H, Chen Z, Cheng Q, Chen R. Forced downregulation of RACK1 inhibits glioma development by suppressing Src/Akt signaling activity. Oncol Rep. 2013; 30: 2195-202. https://doi.org/10.3892/or.2013.2723.
54. Shi S, Deng YZ, Zhao JS, Ji XD, Shi J, Feng YX, Li G, Li JJ, Zhu D, Koeffler HP, Zhao Y, Xie D. RACK1 promotes non-small-cell lung cancer tumorigenicity through activating sonic hedgehog signaling pathway. J Biol Chem. 2012; 287: 7845-58. https://doi.org/10.1074/jbc.M111.315416.
55. De Keersmaecker K, Atak ZK, Li N, Vicente C, Patchett S, Girardi T, Gianfelici V, Geerdens E, Clappier E, Porcu M, Lahortiga I, Luca R, Yan J, et al. Exome sequencing identifies mutation in CNOT3 and ribosomal genes RPL5 and RPL10 in T-cell acute lymphoblastic leukemia. Nat Genet. 2013; 45: 186-90. https://doi.org/10.1038/ng.2508.
56. Altenberg B, Greulich KO. Genes of glycolysis are ubiquitously overexpressed in 24 cancer classes. Genomics. 2004; 84: 1014-20. https://doi.org/10.1016/j.ygeno.2004.08.010.
57. Katayama M, Nakano H, Ishiuchi A, Wu W, Oshima R, Sakurai J, Nishikawa H, Yamaguchi S, Otsubo T. Protein pattern difference in the colon cancer cell lines examined by two-dimensional differential in-gel electrophoresis and mass spectrometry. Surg Today. 2006; 36: 1085-93. https://doi.org/10.1007/s00595-006-3301-y.
58. Tu SH, Chang CC, Chen CS, Tam KW, Wang YJ, Lee CH, Lin HW, Cheng TC, Huang CS, Chu JS, Shih NY, Chen LC, Leu SJ, et al. Increased expression of enolase alpha in human breast cancer confers tamoxifen resistance in human breast cancer cells. Breast Cancer Res Treat. 2010; 121: 539-53. https://doi.org/10.1007/s10549-009-0492-0.
59. Ni S, Zhu J, Zhang J, Zhang S, Li M, Ni R, Liu J, Qiu H, Chen W, Wang H, Guo W. Expression and clinical role of NF45 as a novel cell cycle protein in esophageal squamous cell carcinoma (ESCC). Tumour Biol. 2015; 36: 747-56. https://doi.org/10.1007/s13277-014-2683-5.
60. Kim YJ, Kim BR, Ryu JS, Lee GO, Kim HR, Choi KH, Ryu JW, Na KS, Park MC, So HS, Cho JH, Park DS. HNRNPA1, a Splicing Regulator, Is an Effective Target Protein for Cervical Cancer Detection: Comparison With Conventional Tumor Markers. Int J Gynecol Cancer. 2017; 27: 326-31. https://doi.org/10.1097/IGC.035491R1035491R10868.
61. Liu X, Zhou Y, Lou Y, Zhong H. Knockdown of HNRNPA1 inhibits lung adenocarcinoma cell proliferation through cell cycle arrest at G0/G1 phase. Gene. 2016; 576: 791-7. https://doi.org/10.1016/j.gene.2015.11.009.
62. Tan GS, Lim KH, Tan HT, Khoo ML, Tan SH, Toh HC, Ching Ming Chung M. Novel proteomic biomarker panel for prediction of aggressive metastatic hepatocellular carcinoma relapse in surgically resectable patients. J Proteome Res. 2014; 13: 4833-46. https://doi.org/10.1021/pr500229n.
63. Shin S, Rossow KL, Grande JP, Janknecht R. Involvement of RNA helicases p68 and p72 in colon cancer. Cancer Res. 2007; 67: 7572-8. https://doi.org/10.1158/0008-5472.CAN-06-4652.
64. Kim Y, Kim HD, Youn B, Park YG, Kim J. Ribosomal protein S3 is secreted as a homodimer in cancer cells. Biochem Biophys Res Commun. 2013; 441: 805-8. https://doi.org/10.1016/j.bbrc.2013.10.132.
65. Pogue-Geile K, Geiser JR, Shu M, Miller C, Wool IG, Meisler AI, Pipas JM. Ribosomal protein genes are overexpressed in colorectal cancer: isolation of a cDNA clone encoding the human S3 ribosomal protein. Mol Cell Biol. 1991; 11: 3842-9.
66. McDoniels-Silvers AL, Nimri CF, Stoner GD, Lubet RA, You M. Differential gene expression in human lung adenocarcinomas and squamous cell carcinomas. Clin Cancer Res. 2002; 8: 1127-38.
67. Zhong W, Qin S, Zhu B, Pu M, Liu F, Wang L, Ye G, Yi Q, Yan D. Oxysterol-binding protein-related protein 8 (ORP8) increases sensitivity of hepatocellular carcinoma cells to Fas-mediated apoptosis. J Biol Chem. 2015; 290: 8876-87. https://doi.org/10.1074/jbc.M114.610188.
68. Wu J, Chen XH, Wang XQ, Yu Y, Ren JM, Xiao Y, Zhou T, Li P, Xu CD. ERp19 contributes to tumorigenicity in human gastric cancer by promoting cell growth, migration and invasion. Oncotarget. 2015; 6: 11794-805. https://doi.org/10.18632/oncotarget.3649.
69. Bajić A, Spasić M, Andjus PR, Savić D, Parabucki A, Nikolić-Kokić A, Spasojević I. Fluctuating vs. Continuous Exposure to H_2O_2: The Effects on Mitochondrial Membrane Potential, Intracellular Calcium, and NF-κB in Astroglia. PLoS ONE. 2013; 8: e76383.
70. Erdogan E, Klee EW, Thompson EA, Fields AP. Meta-analysis of oncogenic protein kinase Ciota signaling in lung adenocarcinoma. Clin Cancer Res. 2009; 15: 1527-33. https://doi.org/10.1158/1078-0432.CCR-08-2459.
71. Cunha IW, Carvalho KC, Martins WK, Marques SM, Muto NH, Falzoni R, Rocha RM, Aguiar S, Simoes AC, Fahham L, Neves EJ, Soares FA, Reis LF. Identification of

genes associated with local aggressiveness and metastatic behavior in soft tissue tumors. Transl Oncol. 2010; 3: 23-32.

72. Hutterer E, Asslaber D, Caldana C, Krenn PW, Zucchetto A, Gattei V, Greil R, Hartmann TN. CD18 (ITGB2) expression in chronic lymphocytic leukaemia is regulated by DNA methylation-dependent and -independent mechanisms. Br J Haematol. 2015; 169: 286-9. https://doi.org/10.1111/bjh.13188.

73. Claerhout S, Dutta B, Bossuyt W, Zhang F, Nguyen-Charles C, Dennison JB, Yu Q, Yu S, Balazsi G, Lu Y, Mills GB. Abortive autophagy induces endoplasmic reticulum stress and cell death in cancer cells. PLoS One. 2012; 7: e39400. https://doi.org/10.1371/journal.pone.0039400.

74. Lund RR, Terp MG, Laenkholm AV, Jensen ON, Leth-Larsen R, Ditzel HJ. Quantitative proteomics of primary tumors with varying metastatic capabilities using stable isotope-labeled proteins of multiple histogenic origins. Proteomics. 2012; 12: 2139-48. https://doi.org/10.1002/pmic.201100490.

75. Chocholaty M, Jachymova M, Schmidt M, Havlova K, Krepelova A, Zima T, Babjuk M, Kalousova M. Polymorphisms of the receptor for advanced glycation end-products and glyoxalase I in patients with renal cancer. Tumour Biol. 2015; 36: 2121-6. https://doi.org/10.1007/s13277-014-2821-0.

76. Zhao DC, Lu HW, Huang ZH. Association between the receptor for advanced glycation end products gene polymorphisms and cancer risk: a systematic review and meta-analysis. J BUON. 2015; 20: 614-24.

77. Li T, Qin W, Liu Y, Li S, Qin X, Liu Z. Effect of RAGE gene polymorphisms and circulating sRAGE levels on susceptibility to gastric cancer: a case-control study. Cancer Cell Int. 2017; 17: 19. https://doi.org/10.1186/s12935-017-0391-0.

78. Admoni-Elisha L, Nakdimon I, Shteinfer A, Prezma T, Arif T, Arbel N, Melkov A, Zelichov O, Levi I, Shoshan-Barmatz V. Novel Biomarker Proteins in Chronic Lymphocytic Leukemia: Impact on Diagnosis, Prognosis and Treatment. PLoS One. 2016; 11: e0148500. https://doi.org/10.1371/journal.pone.0148500.

79. Arif T, Vasilkovsky L, Refaely Y, Konson A, Shoshan-Barmatz V. Silencing VDAC1 Expression by siRNA Inhibits Cancer Cell Proliferation and Tumor Growth *In Vivo*. Mol Ther Nucleic Acids. 2014; 3: e159. https://doi.org/10.1038/mtna.2014.9.

80. Shoshan-Barmatz V, Ben-Hail D, Admoni L, Krelin Y, Tripathi SS. The mitochondrial voltage-dependent anion channel 1 in tumor cells. Biochim Biophys Acta. 2015; 1848: 2547-75. https://doi.org/10.1016/j.bbamem.2014.10.040.

81. Grills C, Jithesh PV, Blayney J, Zhang SD, Fennell DA. Gene expression meta-analysis identifies VDAC1 as a predictor of poor outcome in early stage non-small cell lung cancer. PLoS One. 2011; 6: e14635. https://doi.org/10.1371/journal.pone.0014635.

82. Wu M, Tu T, Huang Y, Cao Y. Suppression subtractive hybridization identified differentially expressed genes in lung adenocarcinoma: ERGIC3 as a novel lung cancer-related gene. BMC Cancer. 2013; 13: 44. https://doi.org/10.1186/1471-2407-13-44.

83. Mu Y, Chen Y, Zhang G, Zhan X, Li Y, Liu T, Li G, Li M, Xiao Z, Gong X, Chen Z. Identification of stromal differentially expressed proteins in the colon carcinoma by quantitative proteomics. Electrophoresis. 2013; 34: 1679-92. https://doi.org/10.1002/elps.201200596.

84. Roessler M, Rollinger W, Mantovani-Endl L, Hagmann ML, Palme S, Berndt P, Engel AM, Pfeffer M, Karl J, Bodenmuller H, Ruschoff J, Henkel T, Rohr G, et al. Identification of PSME3 as a novel serum tumor marker for colorectal cancer by combining two-dimensional polyacrylamide gel electrophoresis with a strictly mass spectrometry-based approach for data analysis. Mol Cell Proteomics. 2006; 5: 2092-101. https://doi.org/10.1074/mcp.M600118-MCP200.

85. Oo HZ, Sentani K, Sakamoto N, Anami K, Naito Y, Oshima T, Yanagihara K, Oue N, Yasui W. Identification of novel transmembrane proteins in scirrhous-type gastric cancer by the Escherichia coli ampicillin secretion trap (CAST) method: TM9SF3 participates in tumor invasion and serves as a prognostic factor. Pathobiology. 2014; 81: 138-48. https://doi.org/10.1159/000357821.

86. Chang H, Jeung HC, Jung JJ, Kim TS, Rha SY, Chung HC. Identification of genes associated with chemosensitivity to SAHA/taxane combination treatment in taxane-resistant breast cancer cells. Breast Cancer Res Treat. 2011; 125: 55-63. https://doi.org/10.1007/s10549-010-0825-z.

87. He J, Liu Y, Zhu T, Zhu J, Dimeco F, Vescovi AL, Heth JA, Muraszko KM, Fan X, Lubman DM. CD90 is identified as a candidate marker for cancer stem cells in primary high-grade gliomas using tissue microarrays. Mol Cell Proteomics. 2012; 11: M111 010744. https://doi.org/10.1074/mcp.M111.010744.

88. Lu H, Clauser KR, Tam WL, Frose J, Ye X, Eaton EN, Reinhardt F, Donnenberg VS, Bhargava R, Carr SA, Weinberg RA. A breast cancer stem cell niche supported by juxtacrine signalling from monocytes and macrophages. Nat Cell Biol. 2014; 16: 1105-17. https://doi.org/10.1038/ncb3041.

89. Yan X, Luo H, Zhou X, Zhu B, Wang Y, Bian X. Identification of CD90 as a marker for lung cancer stem cells in A549 and H446 cell lines. Oncol Rep. 2013; 30: 2733-40. https://doi.org/10.3892/or.2013.2784.

90. Yang ZF, Ho DW, Ng MN, Lau CK, Yu WC, Ngai P, Chu PW, Lam CT, Poon RT, Fan ST. Significance of CD90+

cancer stem cells in human liver cancer. Cancer Cell. 2008; 13: 153-66. https://doi.org/10.1016/j.ccr.2008.01.013.

91. Slizhikova DK, Vinogradova TV, Sverdlov ED. [The NOLA2 and RPS3A genes as highly informative markers for human squamous cell lung cancer]. Bioorg Khim. 2005; 31: 195-9.

92. Yu G, Herazo-Maya JD, Nukui T, Romkes M, Parwani A, Juan-Guardela BM, Robertson J, Gauldie J, Siegfried JM, Kaminski N, Kass DJ. Matrix metalloproteinase-19 promotes metastatic behavior *in vitro* and is associated with increased mortality in non-small cell lung cancer. Am J Respir Crit Care Med. 2014; 190: 780-90. https://doi.org/10.1164/rccm.201310-1903OC.

93. Schauer IG, Zhang J, Xing Z, Guo X, Mercado-Uribe I, Sood AK, Huang P, Liu J. Interleukin-1beta promotes ovarian tumorigenesis through a p53/NF-kappaB-mediated inflammatory response in stromal fibroblasts. Neoplasia. 2013; 15: 409-20.

94. Casu B. Structural features and binding properties of chondroitin sulfates, dermatan sulfate, and heparan sulfate. Semin Thromb Hemost. 1991; 17: 9-14.

95. Saini RK, Attarha S, da Silva Santos C, Kolakowska J, Funa K, Souchelnytskyi S. Proteomics of dedifferentiation of SK-N-BE2 neuroblastoma cells. Biochem Biophys Res Commun. 2014; 454: 202-9. https://doi.org/10.1016/j.bbrc.2014.10.065.

96. Li W, Xie C, Yang Z, Chen J, Lu NH. Abnormal DNA-PKcs and Ku 70/80 expression may promote malignant pathological processes in gastric carcinoma. World J Gastroenterol. 2013; 19: 6894-901. https://doi.org/10.3748/wjg.v19.i40.6894.

97. Takahashi RU, Takeshita F, Honma K, Ono M, Kato K, Ochiya T. Ribophorin II regulates breast tumor initiation and metastasis through the functional suppression of GSK3beta. Sci Rep. 2013; 3: 2474. https://doi.org/10.1038/srep02474.

98. Tominaga N, Hagiwara K, Kosaka N, Honma K, Nakagama H, Ochiya T. RPN2-mediated glycosylation of tetraspanin CD63 regulates breast cancer cell malignancy. Mol Cancer. 2014; 13: 134. https://doi.org/10.1186/1476-4598-13-134.

99. Kurashige J, Watanabe M, Iwatsuki M, Kinoshita K, Saito S, Nagai Y, Ishimoto T, Baba Y, Mimori K, Baba H. RPN2 expression predicts response to docetaxel in oesophageal squamous cell carcinoma. Br J Cancer. 2012; 107: 1233-8. https://doi.org/10.1038/bjc.2012.396.

100. Jung HM, Choi SJ, Kim JK. Expression profiles of SV40-immortalization-associated genes upregulated in various human cancers. J Cell Biochem. 2009; 106: 703-13. https://doi.org/10.1002/jcb.22063.

101. Elakoum R, Gauchotte G, Oussalah A, Wissler MP, Clement-Duchene C, Vignaud JM, Gueant JL, Namour F. CARM1 and PRMT1 are dysregulated in lung cancer without hierarchical features. Biochimie. 2014; 97: 210-8. https://doi.org/10.1016/j.biochi.2013.10.021.

102. Mathioudaki K, Scorilas A, Ardavanis A, Lymberi P, Tsiambas E, Devetzi M, Apostolaki A, Talieri M. Clinical evaluation of PRMT1 gene expression in breast cancer. Tumour Biol. 2011; 32: 575-82. https://doi.org/10.1007/s13277-010-0153-2.

103. Bernabe R, Patrao A, Carter L, Blackhall F, Dean E. Selumetinib in the treatment of non-small-cell lung cancer. Future Oncol. 2016; 12: 2545-60. https://doi.org/10.2217/fon-2016-0132.

104. Fahrmann JF, Grapov D, Phinney BS, Stroble C, DeFelice BC, Rom W, Gandara DR, Zhang Y, Fiehn O, Pass H, Miyamoto S. Proteomic profiling of lung adenocarcinoma indicates heightened DNA repair, antioxidant mechanisms and identifies LASP1 as a potential negative predictor of survival. Clin Proteomics. 2016; 13: 31. https://doi.org/10.1186/s12014-016-9132-y.

105. Jia Z, Ai X, Sun F, Zang T, Guan Y, Gao F. Identification of new hub genes associated with bladder carcinoma via bioinformatics analysis. Tumori. 2015; 101: 117-22. https://doi.org/10.5301/tj.5000196.

106. Fukumoto S, Yamauchi N, Moriguchi H, Hippo Y, Watanabe A, Shibahara J, Taniguchi H, Ishikawa S, Ito H, Yamamoto S, Iwanari H, Hironaka M, Ishikawa Y, et al. Overexpression of the aldo-keto reductase family protein AKR1B10 is highly correlated with smokers' non-small cell lung carcinomas. Clin Cancer Res. 2005; 11: 1776-85. doi: 11/5/1776 [pii] 10.1158/1078-0432.CCR-04-1238.

107. Zhu Y, Zhang C, Gu C, Li Q, Wu N. Function of Deubiquitinating Enzyme USP14 as Oncogene in Different Types of Cancer. Cell Physiol Biochem. 2016; 38: 993-1002. https://doi.org/10.1159/000443051 000443051 [pii].

108. Wasylyk C, Zambrano A, Zhao C, Brants J, Abecassis J, Schalken JA, Rogatsch H, Schaefer G, Pycha A, Klocker H, Wasylyk B. Tubulin tyrosine ligase like 12 links to prostate cancer through tubulin posttranslational modification and chromosome ploidy. Int J Cancer. 2010; 127: 2542-53. https://doi.org/10.1002/ijc.25261.

109. Chua HH, Huang CS, Weng PL, Yeh TH. TSGDelta154-1054 splice variant increases TSG101 oncogenicity by inhibiting its E3-ligase-mediated proteasomal degradation. Oncotarget. 2016; 7: 8240-52. https://doi.org/10.18632/oncotarget 6973 [pii].

110. Min SK, Koh YH, Park Y, Kim HJ, Seo J, Park HR, Cho SJ, Kim IS. Expression of HAT1 and HDAC1, 2, 3 in Diffuse Large B-Cell Lymphomas, Peripheral T-Cell Lymphomas, and NK/T-Cell Lymphomas. Korean J Pathol. 2012; 46: 142-50. https://doi.org/10.4132/KoreanJPathol.2012.46.2.142.

111. Xue L, Hou J, Wang Q, Yao L, Xu S, Ge D. RNAi screening identifies HAT1 as a potential drug target in esophageal

squamous cell carcinoma. Int J Clin Exp Pathol. 2014; 7: 3898-907.

112. Hill JJ, Tremblay TL, Fauteux F, Li J, Wang E, Aguilar-Mahecha A, Basik M, O'Connor-McCourt M. Glycoproteomic comparison of clinical triple-negative and luminal breast tumors. J Proteome Res. 2015; 14: 1376-88. https://doi.org/10.1021/pr500987r.

113. Wang HJ, Gao Y, Chen L, Li YL, Jiang CL. RAB34 was a progression- and prognosis-associated biomarker in gliomas. Tumour Biol. 2015; 36: 1573-8. https://doi.org/10.1007/s13277-014-2732-0.

114. Galoian K, Guettouche T, Issac B, Navarro L, Temple HT. Lost miRNA surveillance of Notch, IGFR pathway--road to sarcomagenesis. Tumour Biol. 2014; 35: 483-92. https://doi.org/10.1007/s13277-013-1068-5.

115. Falvella FS, Spinola M, Pignatiello C, Noci S, Conti B, Pastorino U, Carbone A, Dragani TA. AZGP1 mRNA levels in normal human lung tissue correlate with lung cancer disease status. Oncogene. 2008; 27: 1650-6. https://doi.org/10.1038/sj.onc.1210775.

116. Pernemalm M, De Petris L, Eriksson H, Branden E, Koyi H, Kanter L, Lewensohn R, Lehtio J. Use of narrow-range peptide IEF to improve detection of lung adenocarcinoma markers in plasma and pleural effusion. Proteomics. 2009; 9: 3414-24. https://doi.org/10.1002/pmic.200800814.

117. Tran QN. A novel method for finding non-small cell lung cancer diagnosis biomarkers. BMC Med Genomics. 2013; 6: S11. https://doi.org/10.1186/1755-8794-6-S1-S11.

118. Hwang JC, Sung WW, Tu HP, Hsieh KC, Yeh CM, Chen CJ, Tai HC, Hsu CT, Shieh GS, Chang JG, Yeh KT, Liu TC. The Overexpression of FEN1 and RAD54B May Act as Independent Prognostic Factors of Lung Adenocarcinoma. PLoS One. 2015; 10: e0139435. https://doi.org/10.1371/journal.pone.0139435.

119. Shen H, Wang L, Zhang J, Dong W, Zhang T, Ni Y, Cao H, Wang K, Li Y, Wang Y, Du J. ARRB1 enhances the chemosensitivity of lung cancer through the mediation of DNA damage response. Oncol Rep. 2017; 37: 761-7. https://doi.org/10.3892/or.2016.5337.

120. Chen Y, Cui T, Yang L, Mireskandari M, Knoesel T, Zhang Q, Pacyna-Gengelbach M, Petersen I. The diagnostic value of cytokeratin 5/6, 14, 17, and 18 expression in human non-small cell lung cancer. Oncology. 2011; 80: 333-40. https://doi.org/10.1159/000329098.

121. Suo Z, Holm R, Nesland JM. Squamous cell carcinomas. An immunohistochemical study of cytokeratins and involucrin in primary and metastatic tumours. Histopathology. 1993; 23: 45-54.

122. Nakanishi Y, Shimizu T, Tsujino I, Obana Y, Seki T, Fuchinoue F, Ohni S, Oinuma T, Kusumi Y, Yamada T, Takahashi N, Hashimoto S, Nemoto N. Semi-nested real-time reverse transcription polymerase chain reaction methods for the successful quantitation of cytokeratin mRNA expression levels for the subtyping of non-small-cell lung carcinoma using paraffin-embedded and microdissected lung biopsy specimens. Acta Histochem Cytochem. 2013; 46: 85-96. https://doi.org/10.1267/ahc.12024.

123. Tang YN, Ding WQ, Guo XJ, Yuan XW, Wang DM, Song JG. Epigenetic regulation of Smad2 and Smad3 by profilin-2 promotes lung cancer growth and metastasis. Nat Commun. 2015; 6: 8230. https://doi.org/10.1038/ncomms9230.

124. Butler MW, Fukui T, Salit J, Shaykhiev R, Mezey JG, Hackett NR, Crystal RG. Modulation of cystatin A expression in human airway epithelium related to genotype, smoking, COPD, and lung cancer. Cancer Res. 2011; 71: 2572-81. https://doi.org/10.1158/0008-5472.CAN-10-2046.

125. Leinonen T, Pirinen R, Bohm J, Johansson R, Rinne A, Weber E, Kosma VM. Biological and prognostic role of acid cysteine proteinase inhibitor (ACPI, cystatin A) in non-small-cell lung cancer. J Clin Pathol. 2007; 60: 515-9. doi: jcp.2006.038711 [pii] 10.1136/jcp.2006.038711.

126. Yoshida T, Okamoto T, Yano T, Takada K, Kohno M, Suda K, Takenoyama M, Oda Y, Maehara Y. Molecular Factors Associated with Pemetrexed Sensitivity According to Histological Type in Non-small Cell Lung Cancer. Anticancer Res. 2016; 36: 6319-26. https://doi.org/10.21873/anticanres.11228.

127. Song R, Liu Q, Hutvagner G, Nguyen H, Ramamohanarao K, Wong L, Li J. Rule discovery and distance separation to detect reliable miRNA biomarkers for the diagnosis of lung squamous cell carcinoma. BMC Genomics. 2014; 15: S16. https://doi.org/10.1186/1471-2164-15-S9-S16.

128. Tan Q, Wang G, Huang J, Ding Z, Luo Q, Mok T, Tao Q, Lu S. Epigenomic analysis of lung adenocarcinoma reveals novel DNA methylation patterns associated with smoking. Onco Targets Ther. 2013; 6: 1471-9. https://doi.org/10.2147/OTT.S51041.

129. Shi R, Yu X, Wang Y, Sun J, Sun Q, Xia W, Dong G, Wang A, Gao Z, Jiang F, Xu L. Expression profile, clinical significance, and biological function of insulin-like growth factor 2 messenger RNA-binding proteins in non-small cell lung cancer. Tumour Biol. 2017; 39: 1010428317695928. https://doi.org/10.1177/1010428317695928.

130. Szasz AM, Lanczky A, Nagy A, Forster S, Hark K, Green JE, Boussioutas A, Busuttil R, Szabo A, Gyorffy B. Cross-validation of survival associated biomarkers in gastric cancer using transcriptomic data of 1,065 patients. Oncotarget. 2016; 7: 49322-33. https://doi.org/10.18632/oncotarget.10337.

131. Marchi S, Pinton P. The mitochondrial calcium uniporter complex: molecular components, structure and

physiopathological implications. The Journal of Physiology. 2014; 592: 829-39.

132. Luo D, Bu Y, Ma J, Rajput S, He Y, Cai G, Liao DF, Cao D. Heat shock protein 90-alpha mediates aldo-keto reductase 1B10 (AKR1B10) protein secretion through secretory lysosomes. J Biol Chem. 2013; 288: 36733-40. https://doi.org/10.1074/jbc.M113.514877.

133. Klein A, Amigo L, Retamal MJ, Morales MG, Miquel JF, Rigotti A, Zanlungo S. NPC2 is expressed in human and murine liver and secreted into bile: potential implications for body cholesterol homeostasis. Hepatology. 2006; 43: 126-33. https://doi.org/10.1002/hep.20985.

134. Wang Y, Nimec Z, Ryan TJ, Dias JA, Galivan J. The properties of the secreted gamma-glutamyl hydrolases from H35 hepatoma cells. Biochim Biophys Acta. 1993; 1164: 227-35.

135. Bondar OP, Barnidge DR, Klee EW, Davis BJ, Klee GG. LC-MS/MS quantification of Zn-alpha2 glycoprotein: a potential serum biomarker for prostate cancer. Clin Chem. 2007; 53: 673-8. https://doi.org/10.1373/clinchem.2006.079681.

Supplementary Table 1: Summary of current reported or used in the clinic biomarkers for AC and SCC

No	NSCLC reported markers	Marker for: (Ref)
1.	miR21	AC [5]
2.	EGFR - Epidermal growth factor receptor (tyrosine kinase)	Over-expressed in NSCLC [6, 7], AC [8]
3.	ALK-EML4- Tyrosine-protein kinase receptor	AC [9, 10]
4.	ROS1- Proto-oncogene tyrosine-protein kinase ROS	AC [11]
5.	RET- Proto-oncogene tyrosine-protein kinase receptor Ret	AC [12]
6.	c-MET -Hepatocyte growth factor receptor (tyrosine kinase)	Over-expressed in NSCLC [13, 14]
7.	ERBB2- Receptor tyrosine-protein kinase erbB-2	AC [15]
8.	PPP3CA- Serine/threonine-protein phosphatase 2B catalytic subunit alpha isoform. (Mutation)	AC [16]
9.	DOT1L- Histone-lysine N-methyltransferase, H3 lysine-79 specific. (Mutated)	AC [17]
10.	FTSJD1- cap-specific mRNA (nucleoside-2'-O-)-methyltransferase 2. (Mutation)	AC [17]
11.	TTF1-thyroid transcription factor 1	AC [18]
12.	NAPSA- Napsin A	AC [18]
13.	TP63- Tumor protein 63	SCC [19]
14.	p40- ΔNp63	SCC [18, 20]
15.	RASA1- Ras GTPase-activating protein 1	SCC [6, 7]
16.	CD141- Thrombomodulin	SCC [21, 22]
17.	miR205	SCC [5]

The biomarkers used in the clinic are highlighted in grey color. Markers 2 to 6 are predictive marker used to direct targeted therapy and markers 11-14 serve in the clinic for diagnosis of AC or SCC.

Supplementary Table 2: Antibodies used in this study

Antibody	Source and Catalogue Number	Dilution used	
		IHC	WB
Mouse monoclonal anti- β-Actin	Millipore, Billerica, MA, MAB1501	-	1:10,000
Mouse monoclonal anti-ATP5B	Abcam, Cambridge, UK, ab14730	-	1:10,000
Rabbit monoclonal anti-AIF	Abcam, ab32516	1:200	1:1000
Mouse monoclonal anti-Bcl-2	Calbiochem, Nottingham, UK, OP60	-	1:2000
Mouse monoclonal anti-HK-I	Abcam, ab105213	1:500	1:2000
Rabbit monoclonal anti-HK II	Santa Cruz Biotechnology Dallas, TX, sc-27230	-	1:1000
Goat polyclonal anti-LDHA	Epitomics, Cambridge, UK, 1980-1	1:300	1:1000
Rabbit polyclonal anti-MAVS	ALX-210-929-C100		1:2000
Rabbit monoclonal anti-HYOU1	Abcam, ab134944		1:3000
Rabbit monoclonal anti-Hsp60	Abcam, ab46798	-	1:10,000
Mouse monoclonal anti -GAPDH	Abcam, ab9484	-	1:1000
Rabbit monoclonal anti-Rab11b	Santa Cruz Biotechnology, Dallas, TX, ab3612	-	1:1000
Rabbit monoclonal anti-SMAC/ Diablo	Abcam, ab8115	1:300	1:2000
Rabbit monoclonal anti-VDAC1	Abcam, ab15895	1:500	1:5000
Goat anti-Rabbit-HRP	KPL, Gaithersburg, PA, 474-1506	1:250	1:15,000
Donkey anti-Goat-HRP	Abcam, ab97120	-	1:20,000

Antibodies against the indicated protein, their catalogue number, source and the dilutions used in IHC and immunoblot experiments are presented.

Supplementary Table 3: Lung cancer patient characteristics

Patient No.	Age (years)	Gender	Type of Cancer	Stage of Disease
1	76	F	AC	2B
2	77	M	SCC	1A
3	54	M	AC	3A
4	58	M	SCC	2B
5	70	M	SCC	2B
6	69	F	AC	2A
7	36	M	AC	3A
8	62	M	AC	1A
9	82	M	AC	2A
10	48	M	AC	1A
11	65	F	AC	1A
12	78	M	SCC	1B
13	72	M	AC	1A
14	78	M	AC	1A
15	55	M	AC	1A
16	59	F	SCC	1A
17	74	F	SCC	2A
18	65	M	SCC	4
19	76	M	LCC	3A
20	65	F	SCC	1A
21	61	M	AC	1A
22	54	M	SCC	1B
23	56	F	AC	1A
24	58	M	AC	1A
25	55	F	AC	1A
26	76	F	AC	1A
27	85	M	AC	1B
28	55	F	AC	1A
29	62	M	AC	2A
30	79	M	AC	3A
31	81	M	AC	1A
32	62	F	AC	1B
33	83	F	SCC	1B
34	77	M	SCC	1A
35	86	M	SCC	1B
36	74	M	SCC	1B
37	74	M	AC	1A
38	67	F	AC	2A
39	71	M	AC	1A
40	75	F	AC	3A
41	85	M	SCC	1A
42	77	M	SCC	2A
43	59	F	SCC	2A
44	59	F	SCC	1B
45	58	M	AC	1B
46	68	F	AC	1A

Twenty-eight patients were males and twenty-seven were females, with an average age of 68 years (range, 36-86). Disease stage was staged according to the international tumor-node-metastasis system and classified as well (grade 1, n=30), (grade 2, n=10), (grade 3, n=5) (grade 4, n=1).

Supplementary Table 4: qPCR primers used in this study

Gene	Primer sequences
ß-Actin	Forward 5'-ACTCTTCCAGCCTTCCTTCC-3'<br>Reverse 5'- TGTTGGCGTACAGGTCTTTG-3'
AKR1B10	Forward 5'-GAGCAGGACGTGAGACTTCT-3'<br>Reverse 5'-TTTGCCAAGAGGAGACTTCCAA-3'
USP14	Forward 5'-TGCCCTTAAAAGGTATGCAGGT-3'<br>Reverse 5'- TCTCGGCAAACTGTGGGAAA-3'
TTLL12	Forward 5'-TGGAGCACGAGGTTTTCGAC-3'<br>Reverse 5'- CGATGACCTTGTAGCACAGC-3'
TSG101	Forward 5'-GCCAGCTCAAGAAAATGGTGT-3'<br>Reverse 5'- AGGTCTCTGTATTTGTACTGGGT-3'
LRRF2	Forward 5'-CCTCAGCAACAACCCCTCTA-3'<br>Reverse 5'- GGTCATAGATATCCCGCAATTCA-3'
WDR82	Forward 5'-GCTTCGATTTCAGCCCCAAC-3'<br>Reverse 5'- TCTCTTTGGTTTGCCCTCCT-3'
HAT1	Forward 5'-ATGGCGGGATTTGGTGCTAT-3'<br>Reverse 5'- GTTCAATTGCTGTGTTGGTGT-3'

The genes examined, and the forward and reverse sequences of the primers used are indicated.

Supplementary Table 5: Novel bio-markers for selected proteins differentially expressed in healthy donors and lung cancer patients identified by LC-HR MS/MS

See Supplementary File 1

Supplementary Table 6: Differential expression of proteins in AC and SCC

Protein (UniProt accession)	Fold change SCC/AC P-value	Proposed function (cell localization)	Relation to cancer
ITGA7 - Integrin alpha-7 (Q13683)	107.3<br>$1.3x10^{-3}$	Laminin receptor on skeletal myoblasts (Plasma membrane)	Associated with the occurrence and development of bladder cancer [105]
AKR1B10 - Aldo-keto reductase family 1 member B10 (O60218)	17.9<br>$1.9x10^{-3}$	Regulates the balance of retinoic acid and lipid metabolism (lysosome, Secreted)	Potential diagnostic marker specific to smokers' NSCLCs [106]
USP14 - Ubiquitin carboxyl-terminal hydrolase 14 (P54578)	4.4<br>$2.0x10^{-3}$	Proteasome-associated deubiquitinase (Cytosol, Plasma membrane)	Over-expressed in various types of cancer including NSCLC [107]
TTL12 - Tubulin-tyrosine ligase-like protein 12 (Q14166)	8.1<br>$2.1x10^{-3}$	Catalyze post-translational modification of tubulins (Cytosol)	Expression increases during prostate cancer progression to metastasis [108]
TSG101 - Tumor susceptibility gene 101 protein (Q99816)	-38.3<br>$2.6x10^{-3}$	Regulator of vesicular trafficking process (Plasma membrane, Cytosol, Nucleus)	TSG101 splicing variant is linked to progressive tumor-stage and metastasis [109]
LRRFIP2 - Leucine-rich repeat flightless-interacting protein 2 (Q9Y608)	19.3<br>$2.9x10^{-3}$	Positive regulator of the Toll-like receptor (TLR) signaling (Cytosol)	No reported data
WDR82 - WD repeat-containing protein 82 (Q6UXN9)	9.8<br>$3.1x10^{-3}$	Component of histone methyl-transferase complex (Nucleus)	No reported data
HAT1 - Histone acetyltransferase type B catalytic subunit (O14929)	475<br>$4.4x10^{-3}$	Acetylates soluble histone H4, a type B HAT that functions in DNA repair (Nucleus)	High expression in several types of lymphomas. proposed indicator for poor prognosis [110] and potential drug target in esophageal SCC [111]
ACOT1 - Acyl-co-enzyme A thioesterase 1 (Q86TX2)	-2.8<br>$5.6x10^{-3}$	Long chain fatty acid metabolism (Cytosl)	Highly expressed in luminal breast tumors [112]
RAB34 – Ras-related protein Rab-34 (Q9BZG1)	-6.0<br>$7.6x10^{-3}$	GTPase involved in protein transport (Cytosol, Golgi)	A progression- and prognosis-associated biomarker in gliomas [113]. Ras-associated sarcomagenesis [114]

Supplementary Table 7: Potential proteins for distinguishing between AC and SCC. Results were derived from USCS XENA data

Gene	Proposed function (cell localization)	Relation to cancer
	**Higher expression levels in AC**	
**1. AZGP1 - Zinc-alpha-2-glycoprotein (P25311)**	**Lipid degradation in adipocytes, associated with fat losses in some advanced cancers (Plasma membrane, Secreted, Exosomes)**	Associated with AC lung cancer [115]
**2. ACOT1 - Acyl-coenzyme A thioesterase 1 (Q86TX2)**	**Lipid metabolism, long chain fatty acid metabolism (Cytosol)**	**No reported data**
**3. ACAD8 - Isobutyryl-CoA dehydrogenase (Q9UKU7)**	**Acyl-CoA dehydrogenase, catabolism of valine (Mitochondria)**	**No reported data**
**4. NPC2 - Epididymal secretory protein E1 (P61916)**	**Cholesterol transporter (ER, Lysosome, Secreted)**	Associated with lung AC [116]
**5. ABCD3 - ATP-binding cassette sub-family D member 3 (P28288)**	**Involved in fatty acid transport (Peroxisome)**	Associated with lung AC [117]
**6. GALE - UDP-glucose 4-epimerase (Q14376)**	**Galactose metabolism (Cytosol, Exosomes)**	**No reported data**
**7. FEN1 - Flap endonuclease 1 (P39748)**	**Endonuclease involved in DNA replication and repair (Cytosol)**	Associated with lung AC [118]
**8. AKR7A3 - Aldo-Keto Reductase family 7A isoform 3 (O95154)**	**Invoved in Aflotoxin B1 inactivation (Cytosol, Exosome)**	**No reported data**
**9. ARRB1 - Beta-arrestin-1 (P49407)**	**Signaling pathway: Functions in regulating agonist-mediated GPCR (Membrane, Cytosol, Nucleus)**	Enhances chemosensitivity in NSCLC) [119]
**10. RSU1 - Ras suppressor protein 1 (Q15404)**	**Ras signal transduction pathway (Cytosol, Exosomes)**	**No reported data**
**11. LRBA - Lipopoly-saccharide-responsive and beige-like anchor protein (P50851)**	**Coordinates signaling of immune receptors (Cell membrane, ER, Golgi, Lysosome)**	**No reported data**
	**Higher expression levels in SCC**	
**12. Ck5 - Keratin, type II cytoskeletal 5 (P13647)**	**Structural protein (Plasma membrane, Cytosol, Nucleus, Exosome)**	Associated with lung SCC [19, 120]
**13. Ck13 - Keratin, type I cytoskeletal 13 (P13646)**	**Structural protein (Cytosol, Nucleus, Exosome)**	Associated with lung SCC [121]
**14. Ck14 - Keratin, type I cytoskeletal 14 (P02533)**	**Structural protein (Cytosol, Nucleus)**	Associated with lung SCC [120, 122]
**15. Ck17 - Keratin, type I cytoskeletal 17 (Q04695)**	**Structural protein (Cytosol)**	Associated with lung SCC [120]
**16. PFN2 -Profilin-2 (P35080)**	**Structural protein (Cytosol)**	Associated with NSCLC [123]
**17.RANBP1 - Specific GTPase-activating protein (P43487)**	**Signaling pathway, Inhibits GTP exchange on Ran (Cytosol, Nucleus)**	**No reported data**
**18. CSTA - Cystatin-A (P01040)**	**Thiol proteinase inhibitor (Cytosol, Exosomes)**	Associated with lung SCC [124, 125]
**19. GGH - Gamma-glutamyl hydrolase (Q92820)**	**Amino acid metabolism, Hydrolyzes polyglutamate (Lysosome, Secreted)**	Associated with lung cancer NSCLC [126]
**20. RPS6KA3- Ribosomal protein S6 kinase alpha-3 (P51812)**	**Serine/threonine-protein kinase acts downstream of ERK signaling (Nucleus, Cytosol)**	Associated with NSCLC [127, 128]
**21. IGF2BP3-Insulin-like growth factor 2 mRNA-binding protein 3 (O00425)**	**RNA-binding factor that may recruit target transcripts to cytoplasmic protein-RNA complexes (mRNPs)(Nucleus, Cytosol)**	Associated with NSCLC [129]
**22. TIMM44 - Mitochondrial IMM translocase subunit TIM44 (O43615)**	**Mitochondrial peptide transporter, essential component of the PAM complex, ATP binding (Mitochondria)**	**No reported data**
**23. TP63 - Tumor protein 63 (Q9H3D4)**	**Tumor suppressor (Nucleus)**	Associated with lung SCC [19]
**24. SMC2 - Structural maintenance of chromosomes protein 2 (O95347)**	Involved in **condensing chromatin complex** (Cytosol, Nucleus)	**No reported data**

Supplementary Table 8: The relationship between protein expression levels and survival in AC and SCC lung cancer

No	Gene symbol	Half-life (months)				P value	
		AC		SCC		AC	SCC
		High (No.)	low	high	low		
**Higher survival associated with low mRNA level**							
1.	VDAC1	75 (360)	150 (360)	40 (262)	50 (262)	0.0018	0.87
2.	SMAC	65 (360)	120 (360)	60 (262)	60 (262)	7.4×10^{-6}	0.9
3.	HYOU1	65 (360)	115 (360)	60 (262)	60 (262)	2.2×10^{-6}	0.76
4.	TTLL12	75 (337)	120 (336)	50 (135)	55 (136)	2.9×10^{-4}	0.63
5.	RAB34	90 (337)	122 (336)	65 (135)	45 (136)	0.013	0.087
6.	MAVS	90 (337)	115 (336)	65 (135)	50 (136)	0.13	0.25
**Higher survival associated with high mRNA level**							
7.	ARL1	175 (360)	60 (360)	50 (261)	60 (263)	3.4×10^{-14}	0.84
8.	TSG101	137.5 (360)	65 (360)	50 (262)	50 (262)	1.9×10^{-9}	0.18
9.	HAT1	118 (360)	70 (360)	51 (262)	60 (262)	0.0046	0.32
10.	p40	115 (360)	70 (360)	60 (262)	60 (262)	1.4×10^{-7}	0.84
11.	NAPSA	130 (337)	80 (336)	65 (135)	50 (136)	4.2×10^{-5}	0.2
12.	LRRFIP2	120 (361)	75 (359)	52 (261)	52 (263)	0.02	0.51
13.	AIFM1	130 (360)	90 (360)	60 (262)	55 (262)	0.033	0.9
14.	TITF1	127 (360)	81.3 (360)	50 (262)	50 (262)	0.00051	0.11
15.	WDR82	120 (360)	90 (360)	50 (262)	50 (262)	2×10^{-4}	0.95

Data were obtained from KMplot.com. Total sample number was 2437, with initial number in each group presented in parenthesis. The Kaplan–Meier estimator used an earlier (2015) release of the database [130].

Supplementary Table 9: Selected biomarkers for distinguishing between AC and SCC

Protein	SCC/AC		Marker for:
**Method:**	**Proteomics (q-PCR)**	**RNA Seq**	
**1. HAT1 - Histone acetyltransferase type B**	475 (1.8)	1.4	SCC, this study
**2. AKR1B10 - Aldo-keto reductase family 1 member B10 (*secreted*)**	17.9 (20)	4	SCC, this study
**3. USP14 - Ubiquitin carboxyl-terminal hydrolase 14**	4.4 (2)	1.3	SCC, this study
**4. TTLL12 - Tubulin-tyrosine ligase-like protein 12**	8.1 (5)	2.5	SCC, this study
**5. LRRF2 - Leucine-rich repeat flightless-interacting protein 2**	19.3 (3)	1.1	SCC, this study
**6. WDR82 - WD repeat-containing protein 82**	9.8 (4.4)	1	SCC, this study
**7. IGF2BP3 - Insulin-like growth factor 2 mRNA-binding protein**	4.9	1.8	SCC, this study
**8. ITGA7 - Integrin alpha-7 (Q13683)**	107	-1.4	SCC, this study
**9. PFN2 - Profilin-2**	4.9	2.7	SCC, this study
**10. Ck5 - Keratin, type II cytoskeletal 5**	4.6	67.5	SCC [19, 120, 131]
**11. Ck13 - Keratin, type I cytoskeletal 13**	4.4	63.7	SCC [121]
**12. Ck14 - Keratin, type I cytoskeletal 14**	3.2	59.2	SCC [120, 122]
**13. Ck17- Keratin, type I cytoskeletal 17**	2.1	19.5	SCC [120]
**14. GGH Gamma-glutamyl hydrolase *(secreted)***		1.9	SCC
**15. SMAC - Second mitochondria-derived activator of caspases**	**Nuclear localization in SCC**		SCC, this study
**16. TSG101 - Tumor susceptibility gene 101 protein**	-38.3 (-1.6)	-1.1	AC, this study
**17. NAPSA - Napsin A aspartic peptidase**	**-**	**-10**	AC [131]
**18. ACOT1 - Acyl-coenzyme A thioesterase 1**	-2.8	-1.7	AC, this study
**19. RAB34 - Ras-related protein Rab-34**	-6.0	-1.3	AC, this study
**20. FEN1 - Flap endonuclease 1**	3.5	1.5	AC [118]
**21. AZGP1 - Zinc-alpha-2-glycoprotein *(secreted)***	-2.7	-4.1	AC [115]
**22. ACAD8 - Isobutyryl-CoA dehydrogenase, mitochondrial**	-17	-2.4	AC, this study
**23. NPC2 Epididymal secretory protein E1 *(secreted)***		-2.1	AC [116]

Proteins that can be used as biomarkers are presented, with their expression levels in SCC, relative to AD, as determined by proteomics, qPCR (in parenthesis) and RNASeq studies, listed. The source of the data is also indicated. The proteins secreted to the blood are indicated, AKR1B10 [132], NPC2, [133], GGH [134] and AZGP1 [135].
