# Supplementary material for: A molecular signature of lung cancer: potential biomarkers for adenocarcinoma and squamous cell carcinoma
Source: Oncotarget. 2017 Nov 6;8(62):105492–509. doi: 10.18632/oncotarget.22298 (PMC5739654; doi:10.18632/oncotarget.22298)
Supplement: Supplementary file 2 [file oncotarget-08-105492-s002.docx]

**Supplementary Table 5. Novel bio-markers for selected proteins differentially expressed in healthy donors and lung cancer patients identified by LC-HR MS/MS.** Two independent LC-HR MS/MS experiments were performed as described in the Supplementary data section. From each experiment, differentially expressed proteins (p-value <0.01, FC ≥|2**|**) were filtered and proteins differentially expressed in both experiments were selected. Proteins of relevance to lung cancer or with potential as biomarkers are listed. For each protein, the name, fold change and p-value in each experiment, as well as its function, subcellular localization and relevance to cancer, are indicated. Proteins were divided into three groups based on their known association to lung cancer, relation to metabolism or potential as metabolism-related biomarkers for lung cancer**.**

| **No** | **Protein name (Uniprot)** | **Fold change/**  **P value** | **Proposed function**  **(cell localization)** | **Relation to cancer** |
| --- | --- | --- | --- | --- |
| 1 | RB11B - Ras-related protein Rab-11B | >1000  7.7x10^-12^ | Regulator of intracellular membrane trafficking (Extracellular space, Endosome) | Over-expressed in HL-60 leukemia cell line [[23](#_ENREF_23)] |
| 2 | PIGS - GPI transamidase component PIG-S | >1000  1.3x10^-9^ | Component of the GPI transamidase complex (ER) | Over-expressed in breast, ovary and uterus cancers [[24](#_ENREF_24)] |
| 3 | APOOL - Apolipoprotein O-like | >1000  2.1x10^-9^ | Component of a large protein complex that functions in the maintenance of crista junctions (Mitochondria) | **No reported data** |
| 4 | NICA - Nicastrin | >1000  5.8x10^-9^ | A subunit of the gamma-secretase complex (Melanosome) | Regulates breast cancer stem cell properties and tumor growth [[25](#_ENREF_25)] |
| 5 | NDKB - Nucleoside diphosphate kinase B | 14.5  3.1x10^-9^ | Synthesis of nucleoside triphosphates other than ATP (Cytosol, Nucleus) | High expression reduce metastases in breast cancer, melanoma [[26](#_ENREF_26), [27](#_ENREF_27)] |
| 6 | HNRPL - Heterogeneous nuclear ribonucleoprotein L | 7.3  1.3x10^-8^ | Splicing factor, acting as activator or repressor of exon inclusion (Cytosol, Nucleus) | Marker for secondary to brain ALL metastasis [[28](#_ENREF_28)] |
| 7 | LDHA - L-lactate dehydrogenase A chain | 14.8  2.3x10^-8^ | Catalyzes the conversion of pyruvate to lactate and back (Cytosol) | Over-expressed in NSCLC, pancreas, colorectal cancer and more [[29](#_ENREF_29)] |
| 8 | STT3A - Dolichyl-diphospho-oligo saccharide-protein glycosyltransferase | 8.3  1.2x10^-7^ | Catalytic subunit of the N-oligosaccharyl transferase (OST) complex (ER) | Marker for follicular thyroid carcinoma [[30](#_ENREF_30)] |
| 9 | COPA - Coatomer subunit alpha | 14.6  1.3x10^-7^ | Part of a complex that mediates protein transport from the ER to the Golgi, (Cytosol, Golgi) | Associated with mouse mesothelioma progression [[31](#_ENREF_31)] |
| 10 | PDLI5 - PDZ and LIM domain protein 5 | 9.2  1.8x10^-7^ | Z-disc protein that interacts directly with α-actinin-2 (Cytosol, Cell junction) | Associated with gastric cancer [[32](#_ENREF_32)]. High deletion frequencies in oral squamous cell carcinoma [[33](#_ENREF_33)] |
| 11 | HINT1- Histidine triad nucleotide-binding protein 1 | 5.4  2.0x10^-7^ | Hydrolyzes purine nucleotide phosphoramidates  (Cytosol, Nucleus) | Over-expressed in prostate cancer [[34](#_ENREF_34)] |
| 12 | SEC11A - Signal peptidase complex catalytic subunit | >1000  2.3x10^-7^ | Component of a complex that removes signal peptides from proteins translocated into the ER (ER) | Contributes to malignant progression in gastric cancer [[35](#_ENREF_35)] |
| 13 | DDX6 - DEAD box  protein 6 | 62.8  2.5x10^-7^ | Participates in mRNA degradation (Cytosol, Nucleus) | Chromosomal aberrations, DDX6 contribute to lymphomagenesis [[36](#_ENREF_36)] |
| 14 | PGK1 - Phosphoglycerate kinase 1 | 8.9  3.2x10^-7^ | Glycolytic enzyme, converting 3-phospho-D-glycerate to 3-phospho-D-glyceroyl phosphate (Cytosol) | Prognostic biomarker of poor survival and chemoresistance to paclitaxel treatment in breast cancer [[37](#_ENREF_37)] |
| 15 | IF4E - Eukaryotic transltion initiation factor 4E | 7.7  3.5x10^-7^ | Participates in the initiation of translation (Cytosol) | eIF4E over-expression can initiate malignant transformation [[38](#_ENREF_38)] |
| 16 | GDIB - Rab GDP dissociation inhibitor beta | 4.5  3.9x10^-7^ | Regulates the GDP/GTP exchange of most Rab proteins (Cytosol, Plasma membrane) | Increased in metastatic gallbladder cancer cell line SD18H [[39](#_ENREF_39)] and in pancreatic carcinoma [[40](#_ENREF_40)] |
| 17 | RL9 - 60S ribosomal protein L9 | 21.1  4.5x10^-7^ | Translation. Component of the 60S subunit (Cytosol) | Over-expressed in colon adenoma and adenocarcinoma [[41](#_ENREF_41)] |
| 18 | NDUS7 - ADH dehydrogenase (ubiquinone) iron-sulfur protein 7 | >1000  4.7x10^-7^ | Core subunit of the respiratory chain NADH dehydrogenase (Mitochondria) | Amplification in BRCA1-associated ovarian cancer [[42](#_ENREF_42)] |
| 19 | PTBP1 - Polypyrimidine tract-binding protein 1 | 8.4  5.1x10^-7^ | Plays a role in pre-mRNA splicing (Nucleus) | Over-expressed in colorectal cancer [[43](#_ENREF_43)], gemcitabine resistance in pancreatic cancer [[44](#_ENREF_44)], associated with breast tumorigenesis [[45](#_ENREF_45)] |
| 20 | CPNS1 - Calpain small subunit 1 | 11.8  5.7x10^-7^ | Regulatory subunit of the calcium-regulated thiol-protease (Cytosol, Plasma membrane) | Promotes NSCLC progression, over-expressed in liver cancer [[46](#_ENREF_46)], marker of poor prognosis in nasopharyngeal carcinoma [[47](#_ENREF_47)] |
| 21 | PA1B2 - Platelet-activating factor acetyl-hydrolase IB subunit beta | 9.9  5.9x10^-7^ | Inactivates PAF (platelet-activating factor) (Cytosol) | Important in maintaining cancer pathogenicity across a wide spectrum of cancer types [[48](#_ENREF_48)] |
| 22 | PPOX - Proto-porphyrinogen oxidase | >1000  6.6x10^-7^ | Catalyzes the oxidation of protoporphyrinogen-IX to form protoporphyrin-IX (Mitochondria) | Higher expression in faster growing cell lines and primary  colorectal tumors [[49](#_ENREF_49)] |
| 23 | GBLP - Guanine nucleotide-binding protein subunit beta-2-like 1 | 5.8  7.0x10-7 | Intracellular receptor that binds activated PKC (Plasma membrane, Cytosol) | Over-expressed in NSCLC, breast cancer, hepatocellular carcinoma, esophageal squamous cell carcinoma [[46-54](#_ENREF_46)] |
| 24 | RL10 - 60S ribosomal protein L10a | 7.8  7.1x10-7 | Translation. Component of the 60S subunit (Cytosol) | Mutated in T-cell acute lymphoblastic leukemia [[55](#_ENREF_55)] |
| 25 | ENOA - Alpha-enolase | 9.7  7.6x10-7 | Glycolytic enzyme (Cytosol) | Upregulated in lung, brain, breast, colon cancers [[33](#_ENREF_33), [56-58](#_ENREF_56)] |
| 26 | ILF2 - Interleukin enhancer-binding factor 2 | 5.0  7.7x10-7 | Regulatory subunit of complexes involved in mitotic control, DNA break repair, and RNA splicing regulation (Cytosol Nucleus) | Higher expression in esophageal squamous cell carcinoma [[59](#_ENREF_59)] |
| 27 | ROA1 (HNRNPA1) - Heterogeneous nuclear ribonucleo-protein A1 | 5.6  9.3x10-7 | Involved in the packaging of pre-mRNA into hnRNP particles (Cytosol, Nucleus) | Biomarker in cervical carcinoma [[60](#_ENREF_60)], lung cancer progression [[61](#_ENREF_61)] |
| 28 | VPS29- Vacuolar protein sorting-associated protein 29 | 14.7  9.3x10-7 | Component of the retromer cargo-selective complex (CSC) (Cytosol, Cell membrane, Endosome membrane) | **No reported data** |
| 29 | UGPA - UTP-glucose-1-phosphate uridylyltransferase | 7.7  9.5x10-7 | Glucosyl donor in cellular metabolic pathways (Cytosol) | Biomarker for metastatic hepatocellular carcinoma [[62](#_ENREF_62)] |
| 30 | DDX17 - DEAD box protein 17 | 5.6  1.2x10-6 | RNA helicase, involved in transcription and splicing (Nucleus) | Increased expression in colon cancer [[63](#_ENREF_63)] |
| 31 | RS3 - 40S ribosomal protein S3 | 9.4  1.2x10^-6^ | Translation. Component of the 40S subunit (Cytosol, Nucleus) | Proposed as an indicator of malignant tumors [[64](#_ENREF_64)], over-expressed in colorectal cancer [[65](#_ENREF_65)], under-expressed SCC [[66](#_ENREF_66)] |
| 32 | OSBL8 - Oxysterol-binding protein-related protein 8 | >1000  1.2x10^-6^ | Binds 25-hydroxycholesterol and cholesterol (ER membrane, Nucleus membrane) | Down-regulated in hepatoma tissues [[67](#_ENREF_67)] |
| 33 | TXD12 (ERp19) - Thioredoxin domain-containing protein 12 | 37.6  1.4x10^-6^ | Involved in thiol-disulfide oxidase activity (ER) | A thioredoxin-like protein, implicated in development of breast, ovarian, gastrointestinal and gastric cancers [[68](#_ENREF_68)] |
| 34 | USO1 - General vesicular transport factor p115 | 8.7  1.4x10^-6^ | General vesicular transport factor in Golgi (Cytosol, Golgi) | Promotes proliferation of gastric cancer cells [[69](#_ENREF_69)] |
| 35 | COPB2 - Coatomer subunit beta 2 | 12.0  1.4x10^-6^ | Involved in protein transport from the ER to the Golgi (Cytosol, Golgi) | Over-expressed (mRNA) in lung adenocarcinoma tumors [[70](#_ENREF_70)] |
| 36 | SMD3 - Small nuclear ribonucleoprotein Sm D3 | 9.0  1.4x10^-6^ | Core component of the spliceosome (Cytosol, Nucleus) | Associated with metastatic behavior is soft tissue tumors [[71](#_ENREF_71)] |
| 37 | ITB2 - Integrin beta-2 | 5.9  1.5x10^-6^ | Cell adhesion (Plasma membrane, Exosome) | Over-expressed in CLL patients harboring trisomy 12 [[72](#_ENREF_72)] |
| 38 | COPB1 - Coatomer subunit beta 1 | 6.5  1.5x10^-6^ | Involved in protein transport from the ER to the Golgi (Cytosol, Golgi) | Over-expressed in prostate cancer [[73](#_ENREF_73)] |
| 39 | MYH9 - myosin 9 | 6.5  1.7x10^-6^ | Motor protein (Cytosol) | Highly expressed in CL16 breast cancer cell tumors in mice [[74](#_ENREF_74)] |
| 40 | RAGE - Receptor for advanced glycol-sylation end products | -12.2  1.9x10^-6^ | Binds advanced glycation end products (Plasma membrane, Extracellular space) | Polymorphism associated with susceptibility to renal, lung and gastric cancers [[75-77](#_ENREF_75)] |
| 41 | VDAC1 - voltage dependent anion channel 1 | 6.3  2.2x10^-6^ | Channel transporting ions and metabolites, also involved in apoptosis (Mitochondria) | Over-expressed in CLL and lung cancer [[78-80](#_ENREF_78)], predictor of poor outcome in early stage NSCLC [[81](#_ENREF_81)] |
| 42 | ENPL (HSP90B1) - Endoplasmin | 7.3  2.3x10^-6^ | Chaperone that functions in the processing and transport of secreted proteins (ER, Melanosome) | Up-regulated (mRNA) in lung cancer [[82](#_ENREF_82)]. Down-regulated in non-cancer stroma cells from colon cancer tissues [[83](#_ENREF_83)] |
| 43 | CAF17 - Iron-sulfur cluster assembly factor homolog | >1000  2.5x10^-6^ | Involved in the maturation of mitochondrial 4Fe-4S proteins, (Mitochondria) | **No reported data** |
| 44 | PSME3 - Proteasome activator complex subunit 3 | >1000  2.6x10^-6^ | Subunit of the 11S REG proteasome regulator (Cytosol, Nucleus) | Serum tumor marker for colorectal cancer [[84](#_ENREF_84)] |
| 45 | TM9S3 - Transmembrane 9 superfamily member 3 | 11.3  2.6x10^-6^ | Belongs to nonaspanin protein family. Function not known (Plasma membrane, Golgi) | Diagnostic and therapeutic target for scirrhous-type gastric cancer [[85](#_ENREF_85)]. Breast cancer chemoresistance factor [[86](#_ENREF_86)] |
| 46 | THY1 - Thy-1 membrane glycoprotein | 8.5  2.9x10^-6^ | Proposed to function in cell-cell or cell-ligand interactions (Plasma membrane) | Marker for lung, liver, glioma and breast cancer stem cells [[87-90](#_ENREF_87)] |
| 47 | RS3A - 40S ribosomal protein S3a | 11.8  3.3x10^-6^ | Translation, component of the 40S subunit (Cytosol, Nucleus) | Marker for human squamous cell lung cancer [[91](#_ENREF_91)] |
| 48 | MMP19 - Matrix metalloproteinase-19 | >1000  3.3x10-6 | Endopeptidase that degrades various components of the extracellular matrix (ECM) | Involved in NSCLC metastasis and associated with increased mortality [[92](#_ENREF_92)] |
| 49 | ARPC3 - Actin-related protein 2/3 complex subunit 3 | 8.6  4.2x10^-6^ | Component of the Arp2/3 complex involved in regulation of actin polymerization (Cytosol) | Associated with glioma [[93](#_ENREF_93)] |
| 50 | RS15 - 40S ribosomal protein S15 | 15.9  4.3x10^-6^ | Translation, component of the 40S subunit (Cytosol, Nucleus) | RS15 mutations are associated with increased cancer risk [[94](#_ENREF_94)] |
| 51 | PRKDC - DNA-dependent protein kinase catalytic subunit | 10.1  4.5x10^-6^ | Serine/threonine-protein kinase that acts as a molecular sensor for DNA damage (Nucleus) | Highly expressed in advanced neuroblastoma [[95](#_ENREF_95)], associated with gastric carcinoma [[96](#_ENREF_96)] |
| 52 | RPN2 - Ribophorin II | 8.8  4.5x10^-6^ | Protein glycosylation. Essential subunit of the N-oligosaccharyl transferase (OST) complex (ER Plasma membrane) | Breast cancer initiation and metastasis [[97](#_ENREF_97), [98](#_ENREF_98)], associated with docetaxel response in oesophageal SCC [[99](#_ENREF_99)] |
| 53 | RBMX - RNA-binding motif protein, X chromosome | 6.1  4.4x10^-6^ | RNA-binding protein that plays several roles in the regulation of pre- and post-transcriptional processes (Nucleus) | Up-regulated in immortalized cells, cancer cells, and NSCLC tissues [[100](#_ENREF_100)] |
| 54 | ANM1 - Protein arginine N-methyltransferase 1 | 6.2  4.9x10^-6^ | Arginine methyltransferase (Cytosol, Nucleus) | Over-expressed in NSCLC cell lines [[101](#_ENREF_101)], proposed as a marker in breast cancer [[102](#_ENREF_102)] |
| 55 | MAP2K1 (MEK1) - Dual specificity mitogen-activated protein kinase 1 | 165.3  5.7x10^-3^ | A component of the MAP kinase signal transduction pathway, binds extracellular ligands, activates RAS and RAF1 (Cytosol) | Over-expressed in NSCLC [[103](#_ENREF_103)] |
| 56 | EGFR - Epidermal growth factor receptor | 92.6  1x 10^-2^ | Receptor tyrosine kinase binding ligands of the EGF family (Cell membrane, ER, Golgi, Nucleus) | Over-expressed in NSCLC [[6](#_ENREF_6), [7](#_ENREF_7)] |
| 57 | HYOU1- Hypoxia up-regulated protein 1 | -2.7  0.032 | A chaperon molecule belongs to HSP70 family, induced by hypoxia, has cytoprotective activity (ER) | Over-expressed in NSCLC [[104](#_ENREF_104)] |
